# N6-methyladenosine-induced circ1662 promotes metastasis of colorectal cancer by accelerating YAP1 nuclear localization

**DOI:** 10.7150/thno.51342

**Published:** 2021-02-25

**Authors:** Chen Chen, Weitang Yuan, Quanbo Zhou, Bo Shao, Yuying Guo, Weiwei Wang, Shuaixi Yang, Yaxin Guo, Luyang Zhao, Qin Dang, Xiuxiu Yang, Guixian Wang, Qiaozhen Kang, Zhenyu Ji, Jinbo Liu, Zhenqiang Sun

**Affiliations:** 1 Department of Colorectal Surgery, The First Affiliated Hospital of Zhengzhou University, Zhengzhou 450052, Henan, China.; 2 School of Life Science, Zhengzhou University, Zhengzhou, 450001, Henan, China.; 3 Henan Academy of Medical and Pharmaceutical Sciences, Zhengzhou University, Zhengzhou 450052, Henan, China.; 4 School of Basic Medical Sciences, Zhengzhou University, Zhengzhou 450002, Henan, China.; 5 Academy of Medical Sciences, Zhengzhou University, Zhengzhou 450052, Henan, China.; 6 Department of Pathology, The First Affiliated Hospital, Zhengzhou University, Zhengzhou 450052, Henan, China.

**Keywords:** colorectal cancer, N6-methyladenosine (m6A), METTL3, circ1662, YAP1

## Abstract

Tumor metastasis is the leading cause of death in patients with colorectal cancer (CRC). Circular RNAs (circRNAs) have been shown to be involved in cancer progression. However, the regulatory mechanisms of circRNAs involved in CRC tumor metastasis are currently unknown.

**Methods:** High-throughput sequencing was performed on 6 pairs of CRC and adjacent normal tissues to identify the expression profiles of mRNA and circRNA. circ1662 was assessed by RNA-ISH and IHC of a tissue chip. The function of circ1662 in CRC was evaluated by knocking down or overexpressing circ1662. MeRIP-qPCR, RIP-qPCR, and RNA pull-down were performed to determine the relationship between METTL3, circ1662, and YAP1.

**Results:** A novel circRNA, circ1662, exhibited significantly higher expression in CRC tissues than paired normal tissues. High circ1662 expression was correlated with poor prognosis and tumor depth in patients with CRC. Functionally, circ1662 promoted CRC cell invasion and migration by controlling EMT *in vitro* and *in vivo*. Mechanistically, circ1662 directly bound to YAP1 and accelerated its nuclear accumulation to regulate the SMAD3 pathway. Additionally, circ1662 enhanced CRC invasion and migration depending on YAP1 and SMAD3. Interestingly, METTL3 induced circ1662 expression by binding its flanking sequences and installing m6A modifications. Clinically, circ1662 expression strongly correlated with METTL3 and YAP1 protein expression. Moreover, YAP1 expression was negatively correlated with SMAD3 expression.

**Conclusions:** METTL3-induced circ1662 promoted CRC cell invasion and migration by accelerating YAP1 nuclear transport. This result implies that circ1662 is a new prognostic and therapeutic marker for CRC metastasis.

## Introduction

Colorectal cancer (CRC) is a common malignant digestive tract tumor with high morbidity and mortality worldwide 1. Metastasis seriously affects patient survival, and metastasis-related complications are the major cause of patient death 2. Approximately 15-25% of patients with CRC have liver metastasis, which leads to poor prognosis 3. Tumor metastasis refers to the process by which malignant tumor cells continue to expand from the primary site to other parts of the body through lymphatic vessels, blood vessels, or body cavities. Many studies have reported that epithelial-mesenchymal transition (EMT) plays important roles in tumor invasion and metastasis 4, 5. EMT is a common biological process in which endothelial cells are transformed into cells with interstitial phenotype through specific processes, including destabilization of cell junctions. E-cadherin, N-cadherin, vimentin, and tight junction protein 1 (ZO-1) are key proteins regulating EMT. Loss of the protein coded by cadherin 1 (CDH1) gene, E-cadherin, attenuates EMT 6. Moreover, zinc finger E-box-binding homeobox 1 (ZEB1) and SMAD family member 3 (SMAD3) could act as transcription factors to regulate EMT 7. Strategies to accurately control the EMT pathway are potential approaches to control CRC metastasis.

Circular RNA (circRNA) is a class of noncoding RNA with a stable ring structure 8, 9. Some circRNAs are conserved and abundant in eukaryotes, with tissue-specific and cell-specific expression profiles 10. Dysregulated circRNAs have been identified in clinical tumor samples using high-throughput sequencing. Increasing studies have stated that these dysregulated circRNAs play important roles in cancer progression, from tumorigenesis, invasion, and metastasis to colonization 11, 12. Moreover, many circRNAs have been found to be involved in EMT by regulating associated markers, such as E-cadherin, N-cadherin, and vimentin, and related transcription factors, such as those of the Snail, Twist, Slug, and ZEB families 13. For example, increased level of circPRMT5 in urothelial carcinoma of the bladder was found to promote cancer metastasis by regulating E-cadherin, N-cadherin, and vimentin expression 14. circPLEKHM3 overexpression was found to block cancer cell growth, migration, and EMT by reducing EMT-related markers in hepatocellular carcinoma 15. However, the potential mechanisms of how circRNAs regulate EMT in CRC are still unclear and further exploration is needed.

N6-methyladenosine (m6A) is a common RNA modification. It has been reported that m6A modification is mainly involved in splicing, transportation, stabilization, and degradation processes of mRNA and noncoding RNA 16. Recent studies showed that m6A modification occurs in circRNA to control its nuclear transport, degradation, and translation 17. However, further exploration is needed of circRNA processing by m6A modification. In this study, we identified a novel circRNA, circ1662, which is derived from 3 adjacent exons in the yes-associated protein 1 (YAP1) gene, and determined its oncogenic role in CRC. Notably, circ1662 promoted CRC cell invasion and migration by regulating YAP1 protein. We further analyzed circ1662 formation, which was found to be regulated by m6A modification by methyltransferase-like 3 (METTL3) in CRC cells. In addition, METTL3 was found to accelerate CRC metastasis depending on the regulatory mechanism of circ1662. Clinically, circ1662 was found to be positively correlated with METTL3 and YAP1 protein expression. Our findings suggest that circ1662 can be used as a marker to detect cancer metastasis and is a potential therapeutic target in CRC.

## Methods and Materials

### Patient tissue specimens and follow-up

Six paired CRC tissues and their matched adjacent normal tissues were obtained from patients after surgical resection at The First Affiliated Hospital of Zhengzhou University. The clinical data of these tissues are shown in Table S1. Thirty CRC tissues were also obtained from patients after surgical resection. These patients did not undergo any anticancer treatment. A CRC tissue chip was obtained from Shanghai Outdo Biotech (Shanghai, China). All patients signed informed consent forms, and the protocols were approved by the Ethics Committee of The First Affiliated Hospital of Zhengzhou University (2019-KY-423) and Shanghai Outdo Biotech (YB M-05-02).

### Cell culture and transfection

HCT116 cells were obtained from iCell Bioscience Inc. (Shanghai, China) and authenticated by short tandem repeat sequencing before use. SW480 cells were obtained from the Biotherapy Center of The First Affiliated Hospital of Zhengzhou University. All cells were cultured in high-glucose DMEM (HyClone, Logan, Australia) supplemented with 10% fetal bovine serum (Biological Industries, Cromwell, CT, USA) at 37 °C and 5% CO_2_. siRNAs targeting circ1662, METTL3, YAP1, and SMAD3 were designed and synthesized by RiboBio (Guangzhou, China). A circ1662 overexpression vector was constructed from pHBLV (Hanbio Biotechnology, Wuhan, China). This plasmid contains two repeated sequences named 5'circFrame and 3circFrame, which promote circRNA formation through reverse complementation 18. A METTL3 overexpression vector was constructed from pcDNA3.1 (Invitrogen, ThermoFisher Scientific, Carlsbad, CA, USA). Following the manufacturer's instructions, Lipofectamine 3000 (Invitrogen, Thermo Fisher Scientific, Carlsbad, CA, USA) was used for transient transfection of siRNA and transfection of the overexpression vectors.

### Construction of stable cell lines

The lentiviral circ1662 overexpression vectors described above, along with pSPAX2 and pMD2G, were transfected into 293T cells for virus packaging. The concentrated virus was used for subsequent stable transfection into HCT116 cells with LipoFilter Reagent (Hanbio Biotechnology, Wuhan, China). Then, the cells were cultured with puromycin (2 μg/mL) to obtain stable cell lines. In order to construct a double-lentivirus stable cell line, we purchased Lv3-GFP-shMETTL3 plasmid (Genepharm, Shanghai, China) and transferred it with the pSPAX2 and pMD2G plasmids into 293T cells for virus synthesis. Then, the virus supernatant was added into HCT116 cells overexpressing circ1662. After 72h, the cells were cultured with puromycin (2 μg/mL) and sorted by flow cytometry (DxFLEX, Beckman Coulter, CA, USA).

### Animal models

All mouse procedures were approved by the Institutional Animal Care and Use Committee of Zhengzhou University. BALB/c nude mice (4 weeks old) were acquired from Vital River Laboratory (Beijing, China). HCT116 cells with stable circ1662 expression (2 × 10^6^ in 100 µL of PBS) were injected via the tail vein. After 45 days, the mice were sacrificed. The lung metastatic carcinoma specimens were processed into paraffin-embedded sections for subsequent H&E staining and IHC.

### RNA isolation, reverse transcription, and qPCR

Total RNA was isolated with RNAiso Plus reagent (Takala, Dalian, China). Then, RNA was reverse transcribed to cDNA with PrimeScript RT Master Mix Kit (Takala, Dalian, China) and subsequently subjected to RT-qPCR using GoTaq qPCR Master Mix (Vazyme, Nanjing, China) according to the manufacturer's instructions. All primers are listed in Table S3. All data were analyzed and normalized to GAPDH.

### Identification of circRNAs

Total RNA was digested for 15 min at 37 °C after the addition of RNase R (4 U per 2 µg of RNA) (Epicentre Biotechnologies, Madison, WI, USA) to CRC cells. Then, circ1662 and YAP1 were detected by RT-qPCR. In addition, CRC cells were cultured with100 ng/mL actinomycin D (Merck, Darmstadt, Germany) for 12 h, and total RNA was isolated to detect expression of circ1662 and YAP1 by RT-qPCR.

### Cell migration and invasion assays

For the cell migration assay, ~1×10^5^ modified HCT116 and SW480 cells were seeded in transwell chambers with 8-μm pore membranes (Corning, NY, USA). After culturing for 48 h, the migrated cells were fixed and the upper cells were removed by wiping. Then, the migrated cells were stained with Giemsa (Solarbio, Beijing, China). Images of the cells were acquired in four fields using an optical microscope. Similarly, for the invasion assay, ~1×10^5^ modified HCT116 and SW480 cells were seeded in transwell chambers with 8-μm pore membranes (Corning, NY, USA) coated with Matrigel (Corning, NY, USA). The invasive cells were stained and imaged. Then, the number of migrated cell and invasive cells was counted. For wound healing assay, modified CRC cells were seeded in 12-well plates. After the cells were grown to 90% confluence, scratch wounds were formed with a 10 μL plastic pipette tip. The width of the initial scratch was recorded as the 0 h value. Then, the scratch was photographed at 24 or 48 h. The migration area (%) was measured using Image J (National Institutes of Health, Bethesda, MD, USA).

### Nuclear and cytoplasmic extraction

Cells were lysed to isolate the nuclear and cytoplasmic fractions using Nuclear and Cytoplasmic Protein Extraction Kit (Beyotime, Shanghai, China) according to the manufacturer's instructions. The proteins were analyzed by western blot to detect YAP1 expression. The nuclear and cytoplasmic fractions of RNA were extracted with PARIS™ Kit (Invitrogen, Thermo Fisher Scientific, Carlsbad, CA, USA) according to the manufacturer's instructions. Then, the RNAs were analyzed by qPCR to detect circ1662 expression.

### Western blot analysis

Total protein was extracted with RIPA buffer containing PMSF (Solarbio, Beijing, China) and quantified with a BCA kit. Then, the proteins were separated on SDS-PAGE gels and transferred to PVDF membranes (Millipore, Massachusetts, USA). The PVDF membranes were blocked with TBST containing 5% skim milk powder and incubated with anti-YAP (No. 14074), anti-phospho-YAP (Ser127) (No. 13008), and anti-SMAD3 (No. 9523) antibodies from Cell Signaling Technology (MA, USA) and anti-ZO-1 (No. 21773-1-AP), anti-ZEB1 (No. 21544-1-AP), and anti-E-cadherin (No. 20874-1-AP) antibodies from Proteintech (Wuhan, China) at 4 °C overnight. Secondary antibodies were hybridized with the membranes at room temperature for 1 h. A chemiluminescence kit (Absin, Shanghai, China) was used to visualize the membrane.

### RNA *in-situ* hybridization (ISH) and fluorescence *in-situ* hybridization (FISH)

For ISH, the tissue chip was dewaxed, incubated with prehybridization solution, and subsequently hybridized with the circ1662 probe (Servicebio, Wuhan, China). Then, the tissue chip was visualized using DAB. The H-score was determined using Quant Center (3DHISTECH, Budapest, Hungary). For FISH, CRC cells were fixed with 4% paraformaldehyde in PBS for 20 min at room temperature. Hybridization with Cy3-circ1662 probe (GenePharma, Shanghai, China) was performed with a FISH kit (RiboBio, Guangzhou, China) following the manufacturer's instructions. Images were acquired by confocal laser scanning microscopy (Zeiss, Jena, Germany).

### Immunofluorescence (IF) and immunohistochemistry (IHC)

Cells were fixed with 4% paraformaldehyde and permeabilized with 0.1% Triton X-100 in PBS for 10 min. Cells were blocked with 5% BSA for 30 min at 37 °C and incubated with anti-YAP and anti-SMAD3 antibodies overnight at 4 °C. The next day, the cells were washed with PBS, incubated with the corresponding secondary antibody for 30 min at 37 °C, then nuclear stained with DAPI. Fluorescence images were acquired using an fluorescence microscope (Olympus, Tokyo, Japan). A semi-quantitative ISH scoring criterion was used to assess E-cadherin and ZEB1 expression. The staining intensity and the number of positive cells were recorded. More details are provided below. 17. METTL3 and YAP1 were scored using Quant Center (3DHISTECH, Budapest, Hungary).

### RNA pull-down assay

RNA pull-down was performed using a kit (BersinBio, Guangzhou, China). Biotinylated circ1662 probe and negative control probe were designed and synthesized by Genepharm (Shanghai, China). Next, the probes (3 μg) were incubated with 50 μL of streptavidin-coated magnetic beads for 30 min at 25 °C. Cells were lysed by RIP buffer with protease inhibitor cocktail and nucleic acids were removed by addition of 40 μL of agarose beads. The processed lysate was incubated with the streptavidin-coated magnetic beads bound with biotinylated probe for 6 h at 4 °C with rotation. Then, the beads-probe-protein complex was washed with NT2 buffer four times (5 min at 4 °C with rotation). The pulled-down proteins were collected in protein elution buffer for 2 h at 37 °C with rotation and then analyzed by western blot.

### RNA immunoprecipitation (RIP) assay

RIP was performed with Magna RIP RNA-Binding Protein Immunoprecipitation Kit (Millipore, MA, USA) according to the manufacturer's instructions. Magnetic beads were coated with 5 μg of antibodies including anti-immunoglobulin G (IgG) (Millipore, MA, USA), anti-METTL3 (Proteintech, Wuhan, China), and anti-YAP1 (Cell Signaling Technology, MA, USA) for 30 min at room temperature. The antibody-coated magnetic beads were incubated with cell lysate from 2×10^7^ cells overnight at 4 °C. Then, the magnetic beads-protein-RNA complexes were washed 6 times with RIP wash buffer and incubated with proteinase K digestion buffer for 30 min at 55 °C with shaking. RNA was finally extracted by phenol-chloroform RNA extraction methods. Then, the RNA was reverse transcribed using Revert Aid First Strand cDNA Synthesis Kit (Thermo Fisher Scientific, Carlsbad, CA, USA). Interactions between METTL3, YAP1, and circ1662 transcripts were assessed by qPCR and normalized to the input.

### Methylated RNA immunoprecipitation (MeRIP)-qPCR

Total RNA from CRC cells was extracted using miRNeasy Mini Kit (Qiagen, North Rhine-Westphalia, Germany) through stringent DNA digestion with RNase-free DNase Set (Qiagen, North Rhine-Westphalia, Germany). Similar to RIP, magnetic beads (Millipore, Massachusetts, USA) were coated with 5 μg of anti-m6A antibody (No. 202003) (Synaptic Systems, Goettingen, Germany) or anti-IgG for 30 min at room temperature. Then, the antibody-coated beads were incubated with 50 µg of total RNA in RNase-inhibiting immunoprecipitation buffer overnight at 4 °C. After proteinase K digestion, m6A-bound RNA was precipitated by phenol-chloroform RNA extraction methods. m6A enrichment was assessed by qPCR and normalized to the input.

### Statistical analysis

All data were analyzed using Prism 5.0 (GraphPad, San Diego, CA, USA) and expressed as mean ± SEM. Chi-squared tests were performed using SPSS Statistics 21 (IBM, Chicago, IL, USA). Significant differences between two independent groups were evaluated by Student's *t*-test. Correlations were analyzed using Pearson's correlation coefficient(*r*) and two-tailed *P-*values. Survival curves were assessed by log-rank (Mantel-Cox) tests. *P* < 0.05 was considered significant. All experiments were performed at least three times.

## Results

### circ1662 is highly expressed and predicts poor prognosis in patients with CRC

**Figure 1:**
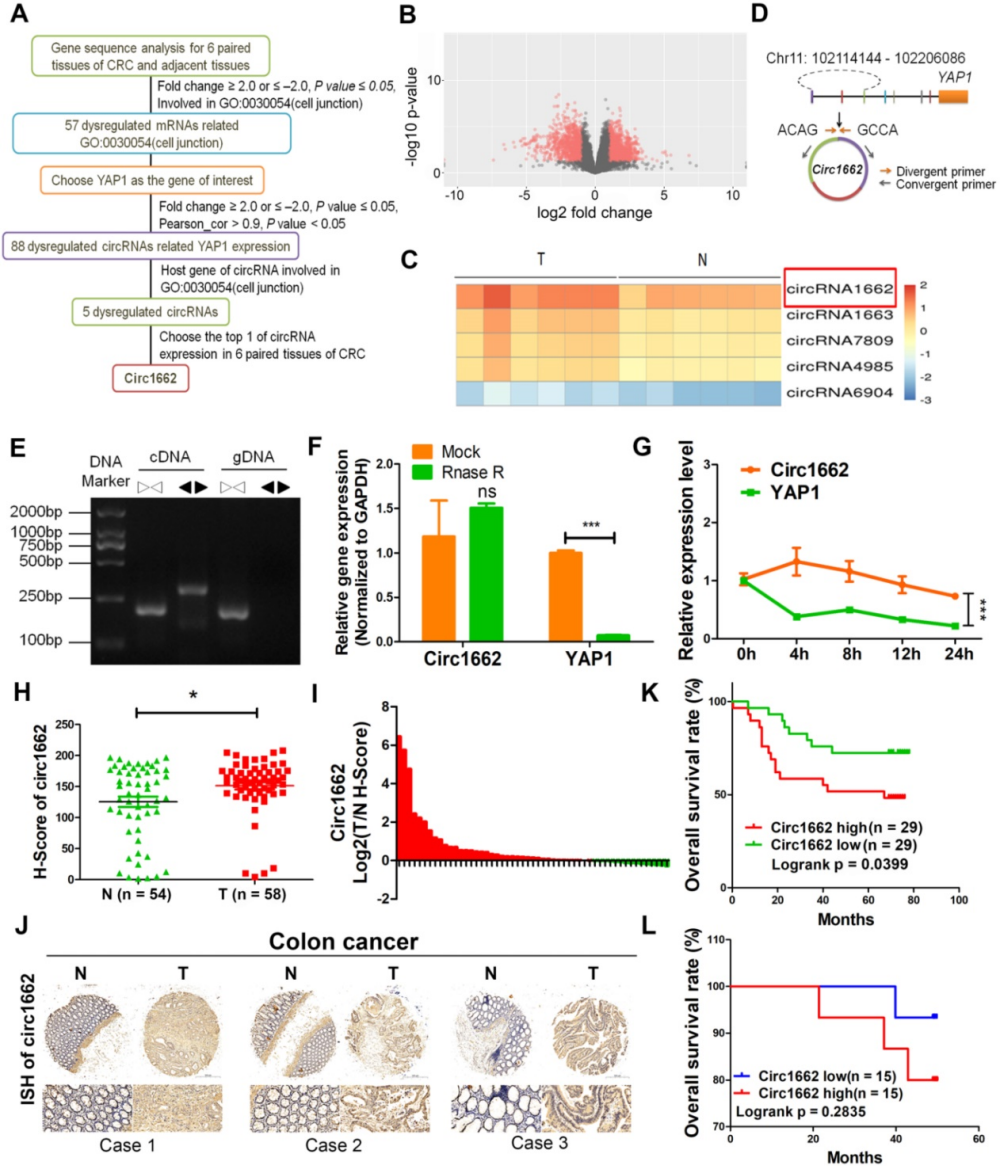
** circ1662 is highly expressed and predicts poor prognosis in patients with CRC.** (A) Flow diagram of circRNAs screening in 6 pairs CRC tissue. (B) The volcano of dysregulated circRNAs in 6 pairs CRC tissue. (C) Heat map for 5 dysregulated circRNAs related with YAP1 and cell junction (GO: 0030054). (D) Circ1662 location information in human genome and primer design. (E) Agarose gel analysis of PCR production using circ1662 divergent primer and convergent primer. (F) qPCR anlaysis of circ1662 and YAP1 expression after Rnase R digestion. (G) qPCR anlaysis of circ1662 and YAP1 expression after SW480 cells culture with Actinomycin D at 0, 4, 8, 12, 24h. (H) H-score of circ1662 in 54 pairs CRC tissues compared to normal tissues from tissue chip. (I) The radio of circ1662 expression in 54 pairs CRC tissues compared to normal tissues from tissue chip. (K) ISH Images of circ1662 in CRC tissue and normal tissues from tissue chip. (J) The overall survival analysis of circ1662 in CRC patient from CRC tissue chip. Log-rank test was use to estimate the significance. (K) The overall survival analysis of circ1662 from 30 CRC patients. GAPDH is used as an internal reference for performing qPCR. Statistical significance was calculated by Student's t test, *P < 0.05, 0.001 < ** P < 0.01, *** P < 0.001, Mean ± SEM.

Many studies have proven that EMT always increases the ability of cancer cells to invade and metastasize by changing single-cell dissemination and cell junctions 19, 20. To explore the mechanisms of EMT in CRC, we analyzed the expression profiles of mRNA from 6 pairs of CRC tissues and adjacent non-tumor tissues by high-throughput sequencing. A total of 1332 mRNAs were upregulated and 795 mRNAs were downregulated in of the CRC tissues compared to their paired non-tumor tissues. We found 57 dysregulated mRNAs (fold change ≥ |2.0| and *P* ≤ 0.05) related to cell junctions (GO: 0030054), which is an important process induced by EMT (Figure 1A). Notably, YAP1, which we previously reported to have a critical role in EMT in CRC, is among these 57 differentially expressed genes 21. However, it is unclear how YAP1 regulation is involved in EMT. EMT-related circRNAs have increasingly been reported to play important roles in various cancers 22. To explore the mechanism of circRNAs-YAP1 in EMT, we also analyzed the circRNA expression profiles of the 6 pairs of CRC tissues and adjacent non-tumor tissues by high-throughput sequencing. A total of 2903 dysregulated circRNAs (1835 upregulated and 1068 downregulated) were identified (Figure 1B). Among these, 88 dysregulated circRNAs were associated with YAP1 expression. Of these circRNAs, 5 have host genes involved with cell junction (GO: 0030054), with circ1662 being the most abundantly expressed in CRC tissues (Figure 1C).

circ1662 is derived from chromosome 11:102114144-102206086 and consists of 3 adjacent exons in the YAP1 gene (Figure 1D). To confirm the ring structure, we designed convergent and divergent primers and determined that circ1662 exists as cDNA rather than gDNA (Figure 1E). Additionally, RNase R digestion and actinomycin D RNA stability assays were performed in CRC cells. After RNase R digestion, the expression of circ1662 was almost unchanged but YAP1 expression reduced significantly (Figure 1F). Similarly, actinomycin D treatment of SW480 cells slightly decreased the half-life of circ1662 compared to that of YAP1, as determined by qPCR (Figure 1G). Collectively, these results suggest that circ1662 has a stable ring structure.

To further verify the expression of circ1662 in CRC tissue, we performed ISH on a tissue chip containing 58 CRC tissues and 54 normal tissues. The H-score determined by Quant Center software indicated higher circ1662 expression in CRC tissues than normal tissues (Figure 1H). Moreover, 70.37% (38/54) of CRC tissues had high circ1662 expression (Figure 1I). The ISH images showed more positive results for circ1662 in CRC tissues than normal tissues (Figure 1J). In addition, high expression of circ1662 in CRC tissues was significantly correlated with tumor depth in patients (Table 1). We analyzed the correlation between circ1662 expression and prognosis by log-rank (Mantel-Cox) test. High circ1662 expression predicted poor overall survival (OS) (Figure 1K). Moreover, we verified that low circ1662 expression predicted better OS in 30 patients with CRC although not statistically significant (Figure 1L). Taken together, these results show that circ1662 is abundant and generally upregulated in CRC tissues and that its expression is correlated with tumor invasion, which is the precursor to cancer metastasis.

### circ1662 promotes CRC cell invasion, migration and EMT

**Figure 2:**
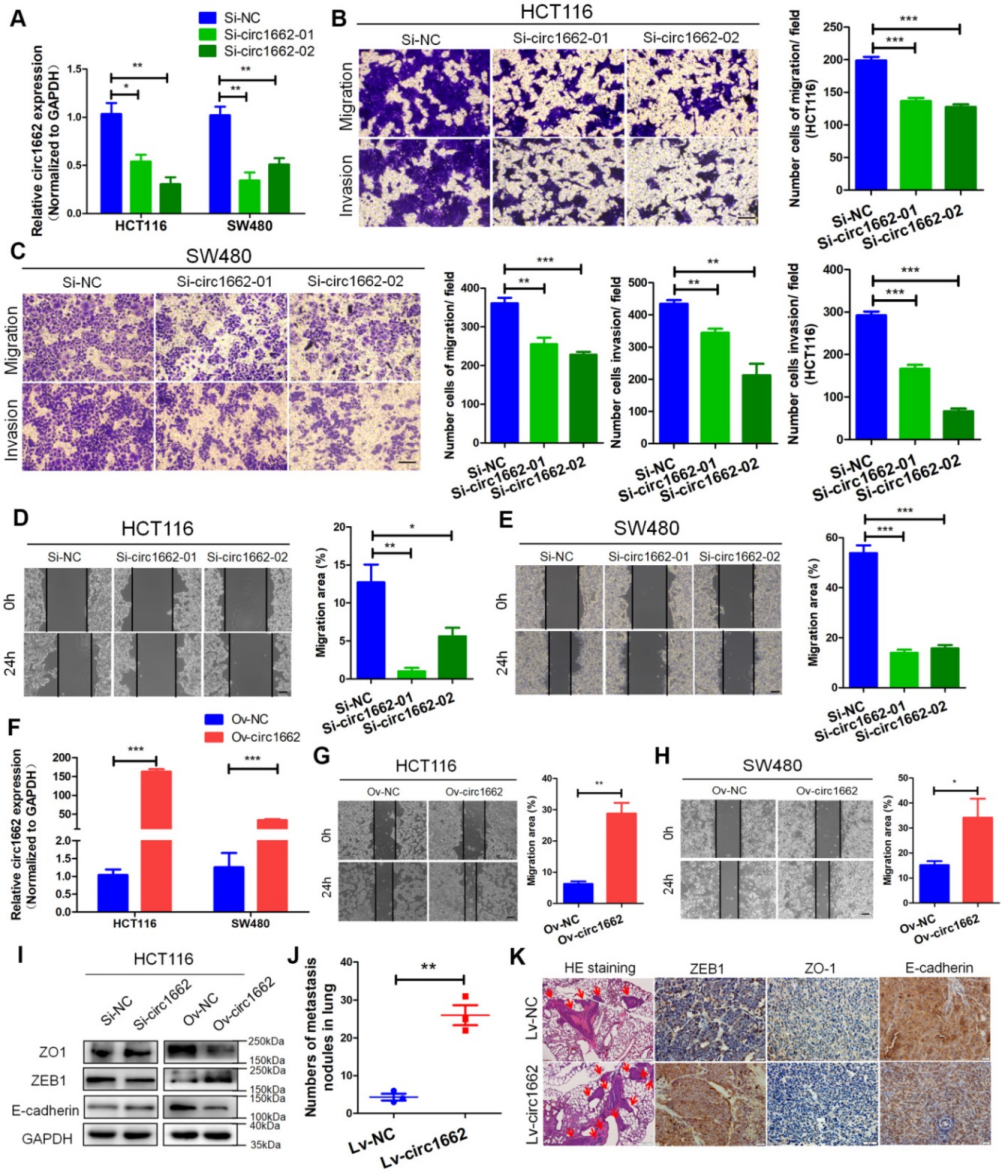
** circ1662 promotes CRC cell invasion, migration and EMT.** (A) qPCR anlaysis of si-circ1662 efficiency in HCT116 and SW480 transfected circ1662 siRNA. (B, C) Transwell assay of HCT116 and SW480 transfected circ1662 siRNA for evaluating migration and invasion. The number of migration and invasion was counted in HCT116 and SW480. (D, E) Wound healing assay of HCT116 and SW480 transfected circ1662 siRNA for imaging at 0, 24h. The migration area was counted by image J in HCT116 and SW480. (F) qPCR anlaysis of overexpressed circ1662 efficiency in HCT116 and SW480 transfected circ1662 vector. (G, H) Wound healing assay of HCT116 and SW480 transfected circ1662 vector for imaging at 0, 24h. The migration area was counted by image J in HCT116 and SW480. (I) Western blot analysis of ZO1, ZEB1 and E-cadherin in HCT116 transfected circ1662 siRNA and circ1662 vector. (J) The number of lung metastasis nodules from Bala/c mice injected stable overexpressed-circ1662 HCT116 via tail vein (n = 3 each group). (K) HE staining and IHC staining of ZEB1, ZO-1 and E-cadherin in metastases from Bala/c mice injected stable overexpressed-circ1662 HCT116 via tail vein. GAPDH is used as an internal reference for performing qPCR. Statistical significance was calculated by Student's t test, *P < 0.05, 0.001 < ** P < 0.01, *** P < 0.001, Mean ± SEM.

To explore the role of circ1662 in CRC cell invasion and migration, we first synthesized two siRNAs that interfere with the motif at the junction sites (Figure S1A). circ1662 expression was significantly decreased in siRNA-transfected CRC cells (Figure 2A). Transwell assay results revealed that inhibiting circ1662 significantly reduced the number of invaded and migrated cells (Figure 2B-C). Wound healing assay results showed a reduced migration area after 24 h in the circ1662 siRNA group compared to the negative control group (Figure 2D-E). In addition, we constructed circ1662 overexpression plasmids to identify the function of circ1662 (Figure S1B). Similar to the above results, circ1662 was substantially overexpressed in CRC cells transfected with the overexpression plasmids (Figure 2F). circ1662 overexpression markedly increased CRC cell invasion and migration (Figure S1C-D). Moreover, circ1662 overexpression accelerated wound healing (Figure 2G-H). We also found that the morphology of HCT116 and SW480 cells tended to be more spindle-shaped in the circ1662 overexpressed groups (Figure S1E). When circ1662 was decreased by siRNA, the expression of key EMT proteins changed, including downregulation of ZEB1 and upregulation of ZO-1 and E-cadherin (Figure 2I). Correspondingly, circ1662 overexpression resulted in ZEB1 upregulation and ZO-1 and E-cadherin downregulation in HCT116 cells (Figure 2I). To explore the oncogenic role of circ1662 *in vivo*, we constructed an HCT116 cell line with stable circ1662 overexpression and injected these cells into BALB/c nude mice via the tail vein (n = 3). After 45 days, we found more lung metastases in the circ1662 overexpression group than the control group (Figure 2J). This result was confirmed by H&E staining (Figure 2K). In addition, IHC revealed decreased expression of ZO-1 and E-cadherin and increased expression of ZEB1 in the circ1662 overexpression group compared with the control group (Figure 2K). Taken together, these results suggest that circ1662 enhances CRC cell invasion, migration, and EMT.

### circ1662 facilitates the invasion and migration of CRC cells by promoting nuclear transport of YAP1

**Figure 3:**
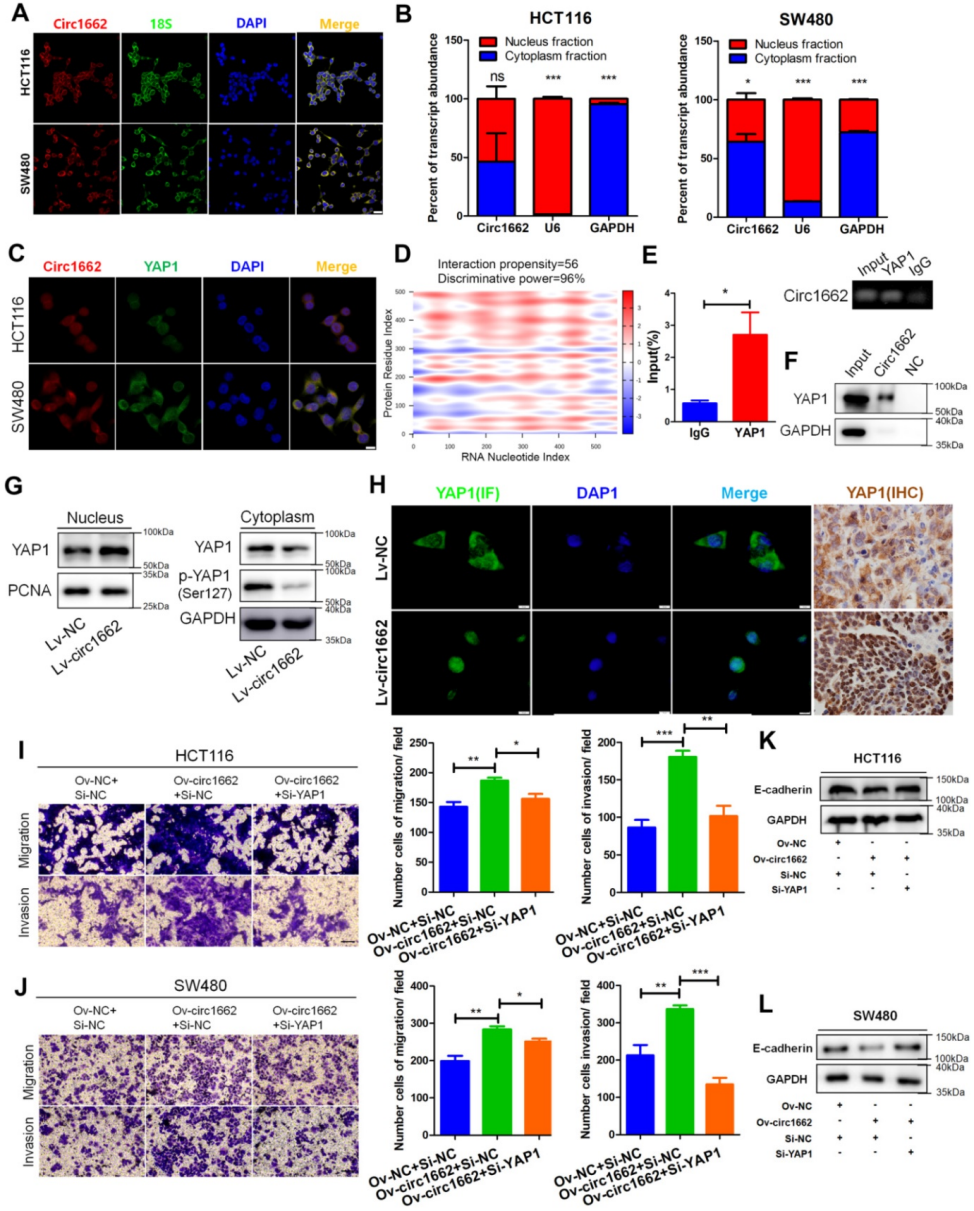
** circ1662 facilitates the invasion and migration of CRC cells by promoting nuclear transport of YAP1.** (A) FISH identified the location of circ1662 in HCT116 and SW480. (B) The nuclear and cytoplasmic fractionation assay assessing the radio of circ1662 in cytoplasmic fractionation in HCT116 and SW480. U6 and GAPDH is used as an internal reference. (C) The colocalization of circ1662 and YAP1 in HCT116 and SW480 by FISH-IF assay. (D) Bioinformatics analysis about potential interaction between circ1662 and YAP1 protein using catRAPID tool. (E) Anti-YAP1 RIP assay pulling circ1662. The result was normalized by input group, IgG group was the negative control. (F) CircRNA pull-down assay performed by biotin-labeled circ1662 probes in HCT116 transfected circ1662 overexpression vector. GAPDH is the negative control. (G) The expression of YAP1 protein in nuclear and cytoplasmic component in stable circ1662-overexpressed HCT116 and its negative control. (H) IF assay in stable circ1662-overexpressed HCT116 cells and IHC assay in metastatic tumor from Bala/c mice injected stable circ1662-overexpressed HCT116 via tail vein. (I) Transwell assay in HCT116 transfected circ1662 vector and YAP1 siRNA. (J) Transwell assay of SW480 transfected circ1662 over-expressed vector and YAP1 siRNA. (K, L) Western blot analysis of E-cadherin regulated by circ1662 and YAP1 in HCT116 and SW480 cells. Statistical significance was calculated by Student's t test, *P < 0.05, 0.001 < ** P < 0.01, *** P < 0.001, Mean ± SEM.

According to our previous report on the important role of YAP1 in EMT 21, we explore the mechanism by which circ1662 regulates YAP1 in CRC. By analyzing Gene Expression Omnibus (GEO) data, we found that YAP1 is upregulated in CRC tissues (Figure S2A). IHC for YAP1 in CRC tissue chips displayed the same result (Figure S2B). We further determined that circ1662 is localized mainly in the cytoplasm but is also localized in the nucleus of CRC cells (Figure 3A). Following nuclear and cytoplasmic fractionation, circ1662 was detected in both fractions by qPCR (Figure 3B). Moreover, a FISH-IF co-localization assay showed similar distributions of circ1662 and YAP1 protein in HCT116 and SW480 cells (Figure 3C). Based on circRNA regulation mechanisms, we hypothesized that circ1662 directly binds to YAP1 protein. This hypothesis was predicted using the catRAPID tool (http://service.tartaglialab.com/page/catrapid_group). Surprisingly, the discriminative power of circ1662 and YAP1 protein binding was 96%, and the interaction propensity was 56 (Figure 3D). RIP-qPCR showed that the YAP1 antibody pulled down circ1662 (Figure 3E). Moreover, we synthesized biotin-labeled probes to target circ1662. These circ1662 probes enriched YAP1 protein in an RNA pull-down assay (Figure 3F). These results suggest that YAP1 can bind circ1662. Many studies have reported that YAP1 can be transported to the nucleus to regulate transcription. 21, 23, 24. However, phosphorylated YAP1 cannot be transported into the nucleus 25. Interestingly, we found that YAP1-Ser127 phosphorylation was reduced when exogenous circ1662 was transfected into HCT116 cells (Figure 3G). Based on the localization of circ1662 in both the cytoplasm and nucleus, we hypothesized that circ1662 may guide YAP1 nuclear transport. circ1662 overexpression increased the nuclear YAP1 protein and decreased the cytoplasmic YAP1 protein (Figure 3G). Subsequent IF assays showed that more YAP1 was present in the nucleus when circ1662 was stably overexpressed in HCT116 cells (Figure 3H). Moreover, we found more nuclear YAP1 in circ1662-overexpressing CRC tumors *in vivo* by IHC (Figure 3H). These results suggest that circ1662 directly binds YAP1 protein and promotes YAP1 nuclear transport. We then determined whether the role of circ1662 in CRC cell invasion and migration is dependent on YAP1. The efficiency of YAP1 siRNA is shown in Figure S1F. Transwell assay results showed that circ1662 overexpression enhanced the number of invaded and metastatic cells and that this effect was weakened by siYAP1 (Figure 3I-J). In addition, exogenous circ1662 reduced E-cadherin expression. This result was reversed by YAP1 siRNA in HCT116 and SW480 cell lines (Figure 3K-L). Taken together, these results indicate that circ1662 promotes CRC invasion and migration by accelerating YAP1 nuclear transport.

### circ1662 inhibits SMAD3 expression via YAP1

**Figure 4:**
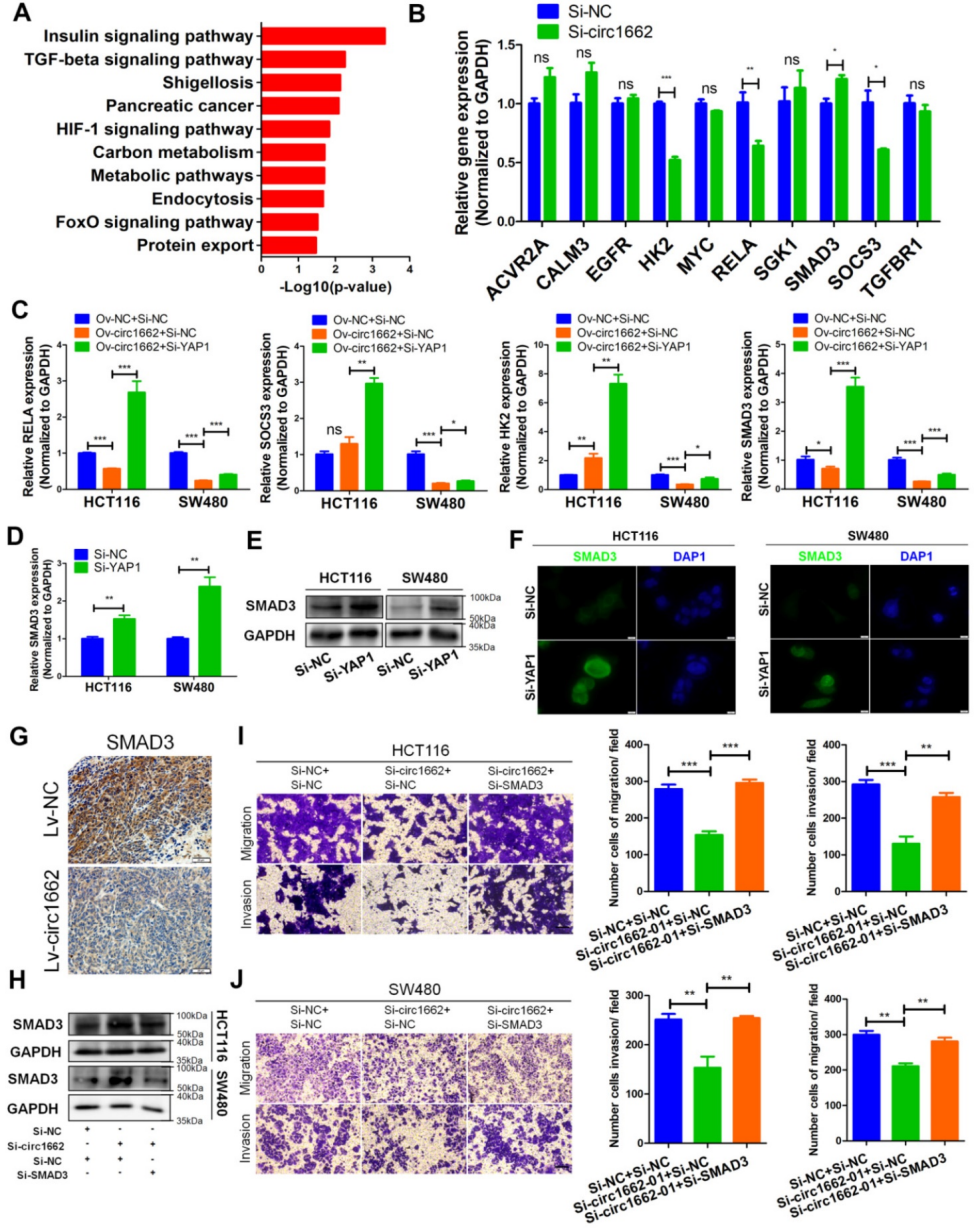
** circ1662 inhibits SMAD3 expression via YAP1.** (A) The top 10 KEGG enrichment analysis of mRNA strongly with YAP1 expression and circ1662 expression in 6 pair colorectal cancer (Pearson correlation coefficient: 0.6-1.0). (B) qPCR anlaysis of ten genes (ACVR2AF, CALM3, EGFR, HK2, MYC, RELA, SGK1, SMAD3, SOCS3, and TGFBR1) screened from the significantly enriched pathways in SW480 transfected circ1662 siRNA. (C) qPCR anlaysis of HK2, SOCS3, RELA and SMAD3 in HCT116 and SW480 transfected circ1662 vector and YAP1 siRNA. (D, E, F) qPCR, Western blot and IF identified SMAD3 mRNA and protein expression level in HCT116 transfected YAP1 siRNA. (G) IHC staining of SMAD3 in metastases from Bala/c mice injected stable overexpressed-circ1662 HCT116 via tail vein. (H) Western blot analysis of SMAD3 expression in HCT116 and SW480 transfected circ1662 siRNA and SMAD3 siRNA. GAPDH is used as an internal reference. (I, J) Transwell assay of HCT116 and SW480 transfected circ1662 siRNA and SMAD3 siRNA. GAPDH is used as an internal reference for performing qPCR. Statistical significance was calculated by Student's t test, *P < 0.05, 0.001 < ** P < 0.01, *** P < 0.001, Mean ± SEM.

To explore the YAP1-regulated mechanism induced by circ1662, we determined the Kyoto Encyclopedia of Genes and Genomes pathways related to YAP1 and circ1662 expression in the 6 pairs of clinical samples. Insulin (hsa04910), TGF-β (hsa04350), HIF-1 (hsa04066), FoxO (hsa04068), MAPK (hsa04010), and Rap1 (hsa04015) were showed in the significantly enriched signaling pathways (Figure 4A). Then, we screened 10 genes from these significantly enriched pathways that were reported to regulate the EMT process: activin A receptor type 2A (ACVR2A), calmodulin 3 (CALM3), epidermal growth factor receptor (EGFR), hexokinase 2 (HK2), MYC, RELA, serum/glucocorticoid-regulated kinase 1 (SGK1), SMAD3, suppressor of cytokine signaling 3 (SOCS3), and transforming growth factor beta receptor 1 (TGFBR1). We found that HK2, RELA, and SOCS3 were downregulated and SMAD3 was upregulated when circ1662 was silenced in SW480 cells (Figure 4B). Moreover, rescue experiments showed that the circ1662 overexpression vector attenuated RELA and SMAD3 expression, which was subsequently inhibited by YAP1 siRNA (Figure 4C). Therefore, we selected SMAD3 as the target of YAP1 regulated by circ1662. To confirm the influence of YAP1 on SMAD3, we performed qPCR, western blotting, and IF. When YAP1 was decreased by siRNA, the SMAD3 mRNA and protein both increased in HCT116 and SW480 cells (Figure 4D-E). IF images also showed stronger fluorescence in the siYAP1 group compared to the negative control group (Figure 4F). Moreover, SMAD3 expression in lung metastases was lower in the circ1662-overexpressing group than in the control group (Figure 4G).

Although many studies have reported that dysregulated SMAD3 is involved in cancer progression, its role in CRC needs further exploration. Therefore, we designed and synthesized siRNA to target SMAD3. The efficiency of SMAD3 siRNA is shown in Figure S1G. Transwell assay results showed that SMAD3 siRNA reduced the numbers of migrated and invaded HCT116 and SW480 cells (Figure S1H-I). Moreover, E-cadherin expression was reduced by SMAD3 siRNA (Figure S1J). Next, we analyzed pan-cancer data of SMAD3 from The Cancer Genome Atlas (TCGA) and found that SMAD3 expression is significantly downregulated in CRC and rectal carcinoma tissues (Figure S2C). GEO data (GSE23878, GSE32323, GSE9348) verified that SMAD3 is downregulated in CRC (Figure S2D). Moreover, high SMAD3 expression is correlated with favorable prognosis in patients with rectal carcinoma (Figure S2E). These results prove that SMAD3 plays a tumor-suppressive role in CRC. To verify the oncogenic role of circ1662 via SMAD3 in CRC, we performed rescue experiments. The results showed that SMAD3 protein expression was increased by circ1662 siRNA and subsequently reduced by SMAD3 siRNA (Figure 4H). The decrease in migrated and invaded cells caused by circ1662 siRNA was mitigated by SMAD3 siRNA (Figure 4I-J). These results suggest that circ1662 promotes CRC cell migration and invasion through YAP1-SMAD3 signaling.

### METTL3 induces circ1662 expression by marking m6A sites in flanking reverse complementary sequences

At present, the mechanism of circRNA formation is unclear. Reverse complementary sequences located in intron flanking sequences of circRNA have been reported to be involved in circRNA formation via backsplicing 26. Cis-regulatory elements located in these reverse complementary sequences, such as Alu and mammalian-wide interspersed repeats (MIRs), can facilitate circRNA formation. We analyzed Alu and MIRs elements in the circ1662 flanking sequences using reverse complementary sequences. By aligning these sequences with the circ1662 flanking sequences in the UCSC Genome Browser (https://genome.ucsc.edu/), we found one MIR element in the antisense strand of the upstream flanking sequence and two Alu elements in the downstream flanking sequence (Figure S3A). These results suggest that regulation of circ1662 formation by reverse complementary sequences may not be dependent on Alu or MIRs elements and may be regulated by other mechanisms.

**Figure 5:**
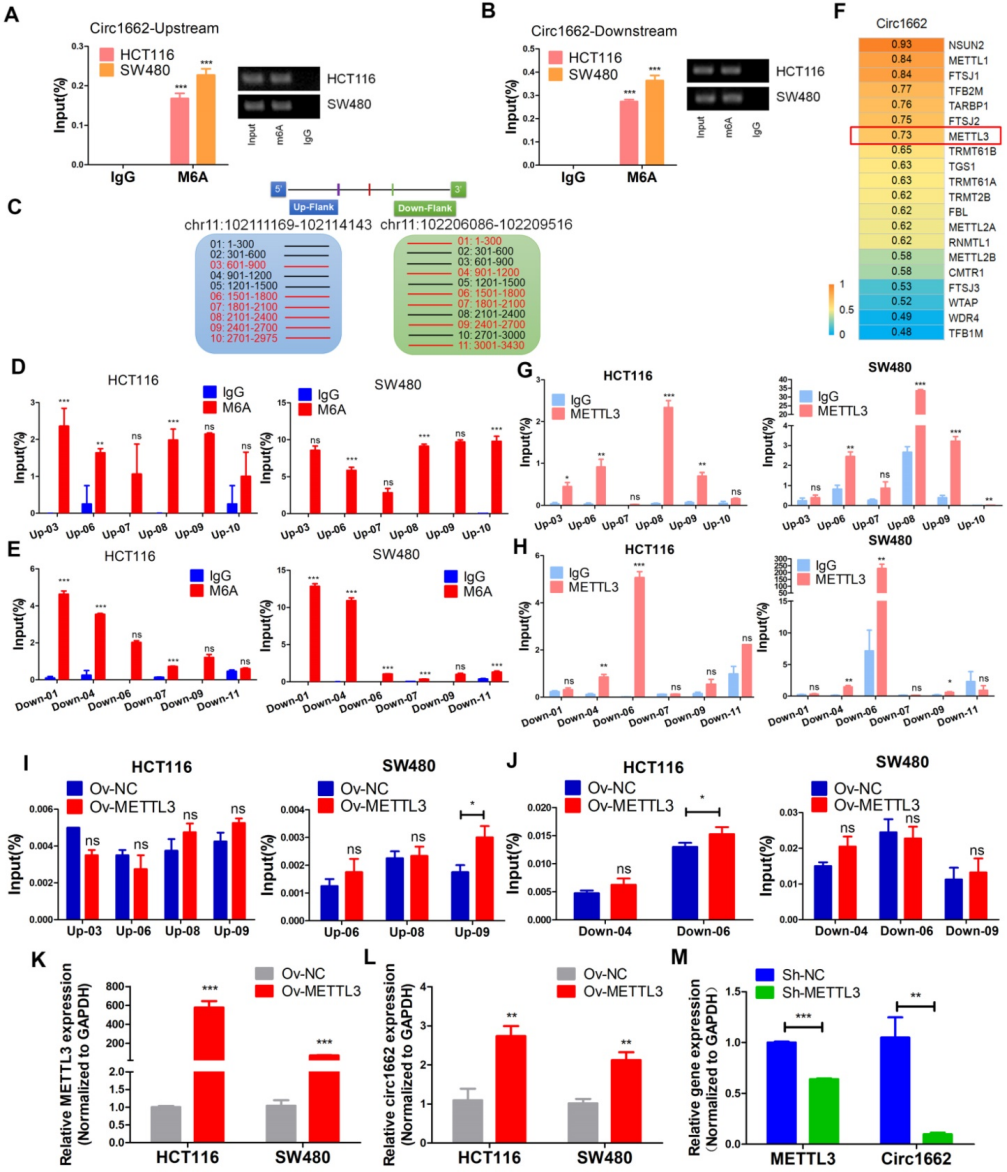
** METTL3 induces circ1662 expression by marking m6A sites in flanking reverse complementary sequences.** (A, B) Circ1662 up-and down flank sequence existed m6A modification using MeRIP-qPCR assay. The qPCR production was identified by agarose gel electrophoresis. The result was normalized by input group, IgG group was the negative control. (C) The flank sequences of circ1662 were divided into 300 nt fragments. The circ1662-up-03, -06, -07, -08, -09, and-10 was marked as red fragements which contain reverse complementary sequences. Circ1662-down-01, -04, -06, -07, -09 and -11 was marked as red fragements which contain reverse complementary sequences. (D, E) MeRIP-qPCR assay detecting circ1662-up-03, -06, -07, -08, -09, -10 and circ1662-down-01, -04, -06, -07, -09 in HCT116 and SW480. The result was normalized by input group, IgG group was the negative control. (F) The correlation analysis between RNA methylation-related molecule and circ1662 in 6 pair colorectal cancer tissues. (G, H) METTL3-RIP-qPCR assay pulling circ1662-up-03, -06, -07, -08, -09, -10 and circ1662-down-01, -04, -06, -07, -09, -11 in HCT116 and SW480. The result was normalized by input group, IgG group was the negative control. (I, J) MeRIP-qPCR assay performing to pull down circ1662-up-03, -06, -08, -09 and circ1662-down-04, -06, -09 in overexpressed-METTL3 HCT116 and SW480. The result was normalized by input group, IgG group was the negative control. (K, L) qPCR analysis of METTL3 expression and circ1662 expression in HCT116 and SW480 cells transfected METTL3-overexpressed vector. (M) qPCR analysis of METTL3 expression and circ1662 expression in HCT116 transfected Sh-METTL3 vector. GAPDH is used as an internal reference for performing qPCR. Statistical significance was calculated by Student's t test, *P < 0.05, 0.001 < ** P < 0.01, *** P < 0.001, Mean ± SEM.

m6A modification is an abundant modification in both mRNA and noncoding RNA 16. Moreover, m6A modification plays an important role in circRNA nuclear transport, degradation and translation 17. However, the mechanism of m6A modification in circRNA formation regulated by reverse complementary sequences has been rarely reported. Early reports found that a large number of m6A modifications exist in nascent RNA, and the splicing site has methylation specificity 27. Therefore, we hypothesized that m6A modification marks are installed in circ1662 reverse complementary sequences to affect circ1662 formation. To verify our hypothesis, we first performed MeRIP-qPCR to measure the m6A level in the reverse complementary sequences of the upstream and downstream flanking sequences of circ1662 in CRC cell lines. After stringent DNA digestion, the MeRIP-qPCR results showed that the upstream and downstream circ1662 flanking sequences exhibited m6A modification (Figure 5A-B). Furthermore, the downstream flanking sequence of circ1662 had a slightly higher m6A level than the upstream flanking sequence. We subsequently blasted the circ1662 upstream flanking sequence against its downstream flanking sequence using the Blast tool (https://blast.ncbi.nlm.nih.gov/Blast.cgi). Interestingly, these flanking sequences contained 10 pairs of short reverse complementary sequences (Table S2). Next, the upstream and downstream flanking sequences were divided into 300 nt fragments each, and specific primers for every fragment were designed and synthesized. Among these fragments, reverse complementary sequences were located in 1662-up-03, -06, -07, -08, -09, and-10 and 1662-down-01, -04, -06, -07, -09, and-11 (Figure 5C). We further identified m6A modification of 1662-up-03, -06, -07, -08, -09, and-10 and 1662-down-01, -04, -06, -07, -09, and-11 in HCT116 and SW480 cells. Among the upstream flanking sequence fragments, 1662-up-03, -06, -08, and-09 in HCT116 cells and 1662-up-03, -06, -08, -09, and-10 in SW480 cells had higher levels of m6A modification than the other fragments (Figure 5D). Moreover, among the downstream flanking sequence fragments, m6A modification was identified on 1662-down-01, -04, -06, -07, and-09 in HCT116 cells and 1662-down-01, -04, -06, -07, -09, and-11 in SW480 cells (Figure 5E). These sequences were identified by nucleic acid gel electrophoresis (Figure S3B).

To determine the key regulator of m6A modification in circ1662 regulation, we analyzed the correlation between circ1662 and mRNAs related to RNA methylation (GO:0001510) in our sequencing data. A total of 27 RNA methylation-related mRNAs were identified and the top 20 are shown in Figure 5F. METTL3 was selected due to its critical role in m6A modification. Next, an RIP assay using anti-METTL3 antibody was implemented to determine the binding motif in the reverse complementary sequences of the circ1662 flanking sequences. The fragments1662-up-03, -06, -08, and -09 in HCT116 and 1662-up-06, -08, -09, and-10 in SW480 were pulled down by METTL3 antibodies significantly more than by IgG (Figure 5G). Moreover, the fragments1662-down-04 and -06 in HCT116 and 1662-down-04, -06, and -09 in SW480 were pulled down by METTL3 antibodies significantly more than by IgG (Figure 5H). These sequences were identified by nucleic acid gel electrophoresis (Figure S3C). These results indicate that METTL3 can significantly bind to circRNA flanking inverted repeats. To prove the role of METTL3 in circ1662 formation via m6A modification, we extracted RNA from METTL3-overexpressing CRC cells and corresponding negative control CRC cells and performed MeRIP-qPCR. The results showed that more 1662-up-09 was pulled down from METTL3-overexpressing CRC cells, especially SW480 cells, than from the corresponding negative control cells (Figure 5I). Moreover, 1662-down-06 was significantly overexpressed in HCT116 cells (Figure 5J). In addition, we assessed circ1662 expression in cells transfected with METTL3 overexpression plasmid and shMETTL3 plasmid. As expected, METTL3 overexpression markedly induced circ1662 expression in both the HCT116 and SW480 cell lines (Figure 5K-L). Correspondingly, circ1662 was downregulated by shMETTL3 plasmid in HCT116 cells (Figure 5M). Taken together, these results imply that METTL3 induces circ1662 expression by installing m6A modifications in circ1662 flanking reverse complementary sequences.

### METTL3 promotes CRC cell invasion and migration via the circ1662-YAP1-SMAD3 axis

**Figure 6:**
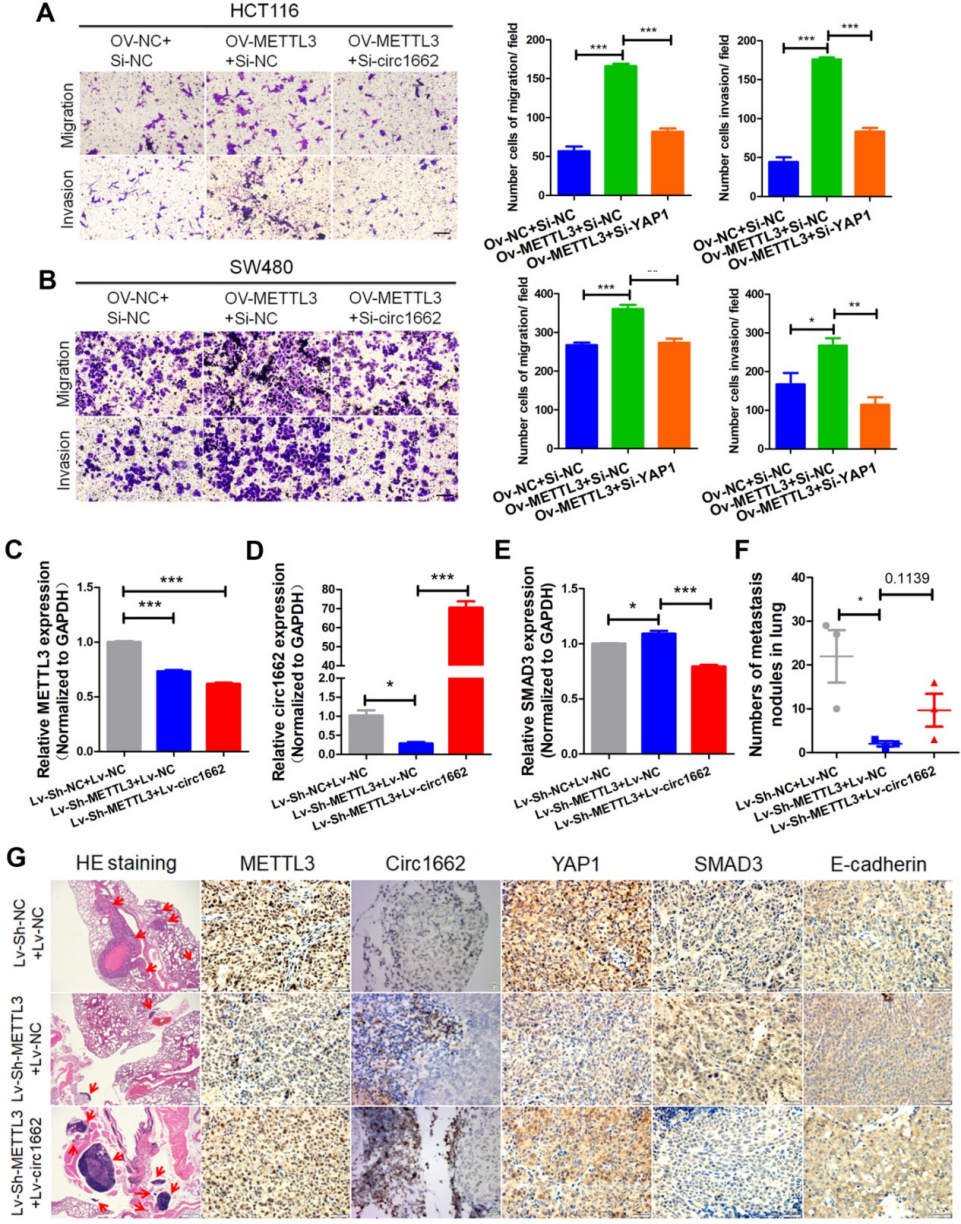
** METTL3 promotes CRC cell invasion and migration via the circ1662-YAP1-SMAD3 axis.** (A, B) Rescues of transwell assays when HCT116 and SW480 cells were transfected METTL3-overexpressed vector and subsequent circ1662 siRNA. (C, D) The efficiency of METTL3 and circ1662 when constructing double-lentivirus transfected HCT116 using qPCR, GAPDH is the negative control. (E) The expression of SMAD3 in double-lentivirus transfected HCT116 using qPCR, GAPDH is the negative control. (F) The number of lung metastasis nodules from Bala/c mice injected stable double-lentivirus HCT116 via tail vein (n = 3 each group). (K) HE staining, ISH staining of circ1662 and IHC staining of METTL3, YAP1, SMAD3 and E-cadherin in lung metastases from Bala/c mice injected stable double-lentivirus HCT116 via tail vein. GAPDH is used as an internal reference for performing qPCR. Statistical significance was calculated by Student's t test, *P < 0.05, 0.001 < ** P < 0.01, *** P < 0.001, Mean ± SEM.

METTL3 has been reported to act as an oncogene in multiple cancers 28. Using UALCAN (http://ualcan.path.uab.edu/) to analyze data from TCGA, we found that METTL3 is upregulated in CRC and rectal carcinoma samples compare to normal tissue samples (Figure S4A). We verified this finding by IHC of CRC tissue chips. The IHC images showed more positive METTL3 expression in CRC tissues than in normal tissues (Figure S4B). Additionally, a high H-score for METTL3 was found in CRC tissue (Figure S4C) and high METTL3 expression was correlated with poor prognosis (Figure S4D). To explore the role of METTL3, we performed wound healing and transwell assays. The transfection efficiency of METTL3 siRNA is shown in Figure S1K. Silencing METTL3 weakened the wound healing ability of HCT116 and SW480 cells (Figure S4E-G) and decreased CRC cell invasion and migration (Figure S4F-H). Moreover, the results of rescue experiments showed that circ1662 dysregulation reduced the inhibitory effect of METTL3 overexpression on CRC cell invasion and migration (Figure 6A-B). To further explore the regulation of circ1662 and its downstream molecules by METTL3, we constructed three groups of double-lentivirus stably transfected HCT116 cells. When METTL3 expression was knocked down, the expressions of METTL3 and circ1662 significantly decreased, while the expression of SMAD3 significantly increased. After overexpression of circ1662 in the METTL3 knockdown group, METTL3 expression remained significantly decreased, circ1662 expression was significantly increased, but SMAD3 expression was significantly decreased (Figure 6C-E). Then, we injected the three groups of cells into BALB/c nude mice via the tail vein. After 45 days, we counted the number of lung metastases and detected expression of METTL3, circ1662, and its downstream molecules. The number of lung metastases was obviously reduced in the Lv-sh-METTL3 group compared to the control group, which was subsequently increased by circ1662 overexpression lentivirus (Figure 6F). ISH and IHC assays revealed decreased expression of METTL3, circ1662, and YAP1, and increased expression of SMAD3 and E-cadherin in the Lv-sh-METTL3 group compared to the control group (Figure 6G). When circ1662 was overexpressed in the Lv-sh-METTL3 group, YAP1, SMAD3, and E-cadherin expression showed opposite trends, but METTL3 expression did not significantly change (Figure 6G). Taken together, these data suggest that METTL3 facilitates CRC cell invasion and migration through the circ1662-YAP1-SMAD3 axis.

### Clinical correlations among METTL3, circ1662, YAP1 and SMAD3 in CRC

**Figure 7:**
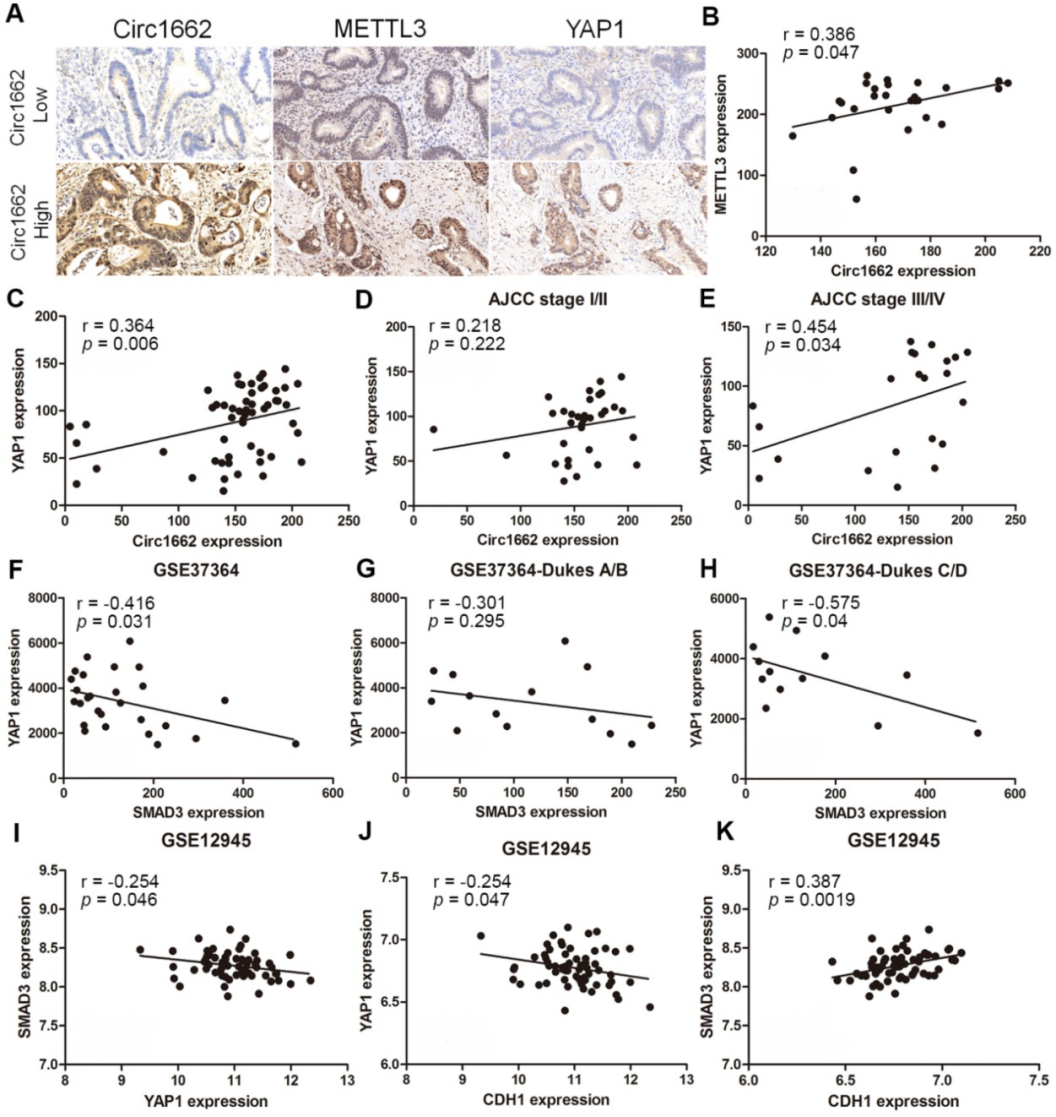
** Clinical correlations among METTL3, circ1662, YAP1 and SMAD3 in CRC.** (A) ISH Image of circ1662, IHC of METTL3 and YAP1 in CRC tissue chip. (B) Correlation analysis between circ1662 and METTL3 in CRC tissue chip (n = 27). (C) Correlation analysis between circ1662 and YAP1 in CRC tissue chip (n = 55). (D) Correlation analysis between circ1662 and YAP1 in the tissue of AJCC stage I/II from CRC tissue chip (n = 33). (E) Correlation analysis between circ1662 and YAP1 in the tissue of AJCC stage III/IV from CRC tissue chip (n = 22). (F) GEO data (GSE37364) analyzing the correlation between YAP1 and SMAD3 in CRC tissues (n = 27). (G) GEO data (GSE37364) analyzing the correlation between YAP1 and SMAD3 expression in Dukes A/B of CRC tissues (n = 14). (H) GEO data (GSE37364) analyzing the correlation between YAP1 and SMAD3 expression in Dukes C/D of CRC tissues (n = 13). (I) GEO data (GSE12495) analyzing the correlation between YAP1 and SMAD3 in CRC tissues (n = 62). (J, K) Correlation analysis between CDH1 and YAP1, CDH1 and SMAD3 from GEO data (GSE12495) in CRC tissues (n = 62).

**Figure 8:**
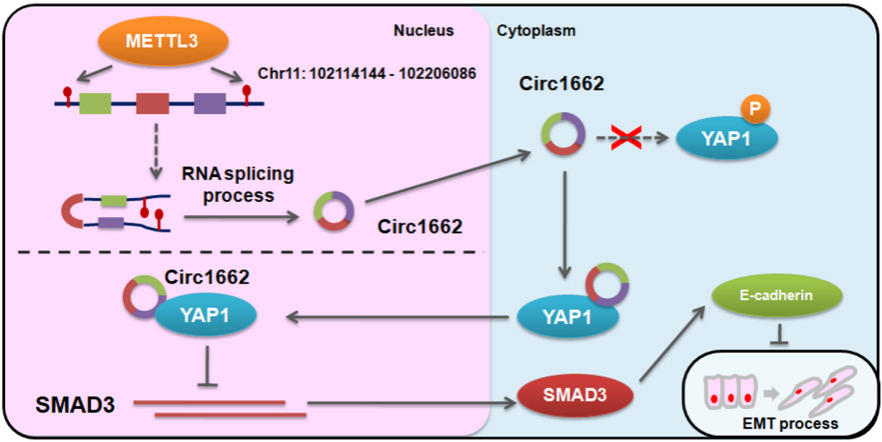
Digrams of the proposed mechanism in this study.

Based on the regulatory mechanism linking METTL3, circ1662, YAP1, and SMAD3, we further analyzed the clinical relevance of this relationship. ISH images ofcirc1662 and IHC images of METTL3 and YAP1 clearly showed that high circ1662 expression was positively correlated with high YAP1 expression and high METTL3 expression in CRC tissue chips (Figure 7A-C). Moreover, we found that YAP1 was significantly positively correlated with circ1662 in CRC tissue chips of American Joint Committee on Cancer (AJCC) stage III-IV but not ACJJ stage I-II (Figure 7D-E). In addition, GEO data analysis results showed that YAP1 expression was negatively correlated with SMAD3 expression in CRC tissues (Figure 7F, 7I, S2F-G). Similarly, YAP1 expression was significantly negatively correlated with SMAD3 expression in CRC tissues of Dukes C/D (Figure 7G-H). Moreover, CDH1 expression was significantly negatively correlated with YAP1 expression (Figure 7J). However, SMAD3 expression was significantly positively correlated with CDH1 expression (Figure 7K). As shown in Figure 8, we showed that circ1662 is induced by METTL3 via m6A modification. Upregulated circ1662 directly binds YAP1 protein and promotes its nuclear transport. Then, YAP1 inhibits SMAD3. In brief, METTL3-induced circ1662 promotes EMT, accelerating CRC metastasis via the YAP1-SMAD3 signaling pathway.

## Discussion

EMT has emerged as a central driver of tumor metastasis, which leads to poor prognosis in patients with CRC 29, 30. In monolayer culture, polygonal epithelial cells are usually polarized along their apical axis and are tightly connected to each other through adhesion and tight junctions 31. Therefore, cell-cell connections are an indispensable part of EMT. Through high-throughput sequencing, we identified57 dysregulated mRNAs related to cell connection (GO:0030054) in 6 pairs of CRC and adjacent non-tumor tissues. Notably, YAP1, which we previously reported to have a critical role in EMT of CRC cells, is among these 57 differentially expressed genes 21. However, the upstream mechanism of YAP1 participation in EMT is unclear. Many studies have shown that circRNAs play important roles in EMT and regulate cancer progression 32, 33. In urothelial carcinoma of the bladder, circPRMT5 induced EMT and promoted metastasis by sponging miR-30c 32. In non-small cell lung cancer (NSCLC), circPTPRA sponged miR-96-5p to inhibit EMT and hamper metastasis 33. Therefore, we speculated that circRNA may affect EMT in CRC by regulating YAP1. To explore the mechanism of circRNAs-YAP1 in EMT, we analyzed the circRNA expression profiles in6 pairs of CRC tissues and adjacent non-tumor tissues and paid attention to the function and regulation mechanism of circ1662.

Our findings proved that circ1662 is upregulated in CRC tissue and that its high expression predicts poor prognosis in patients with CRC. Moreover, circ1662 expedites the EMT process *in vitro* and *in vivo*. We further asked how circ1662 regulates YAP1. The general mechanisms underlying circRNA regulation are its action as an miRNA/protein “sponge” or its translation into apeptide 34. According to current reports, two hypotheses are possible: circ1662 regulates YAP1 molecules through miRNA, or circ1662 directly binds to YAP1 molecules. To ensure regulation of YAP1 by circ1662, we performed a circRNA localization assay. The results showed that circ1662 exists in both the cytoplasm and nucleus, and there is no difference in these localizations. Moreover, FISH-IF co-localization assay results showed that both YAP1 protein and circ1662 exist in the cytoplasm and nucleus, and the fluorescence distribution of YAP1 protein overlaps that of circ1662. Therefore, we hypothesized that circ1662 and YAP1 protein may directly bind, which was subsequently confirmed by bioinformatics prediction, RIP assay and circRNA pull-down assay. Host genes and circRNAs have different relationships because circRNAs and their host genes maintain a dynamic balance under homeostasis. At present, few circRNAs have been proven to regulate their homologous proteins. cia-cGAS in hematopoietic stem cells (HSCs) is one of these. This circRNA bound the DNA sensor cGAS in the nucleus, blocking its synthase activity, to maintain the balance between HSC self-renewal and differentiation 35. However, this equilibrium was destroyed by tumorigenesis. circFoxo3 is located in the cytoplasm and induces cancer cell apoptosis by inhibiting ubiquitylation of FOXO3. 36. Consistent with these studies, we show that circ1662 is positively correlated with YAP1 in CRC. Overexpressed circ1662 promotes YAP1 protein transport to the nucleus and reduces the YAP1 protein level in the cytoplasm. Moreover, circ1662 overexpression decreases the level of p-YAP1, which cannot be transported into the nucleus 37. It is well known that there are 24 phosphorylation sites in YAP1. It is currently unknown if circ1662 binds to YAP1 phosphorylation sites to prevent its phosphorylation. In the future, we will identify which YAP1 phosphorylation site(s) circ1662 binds to. Rescue experiments showed that circ1662 promotes EMT in colon cancer depending on YAP1 protein. Collectively, this evidence suggests that circ1662 can directly bind YAP1 and promote its nuclear transport to accelerate CRC progression.

YAP1 is the key downstream effector of the Hippo pathway and a transcriptional regulator that plays important roles in multiple cancers 38, 39. In a previous study, we proved that upregulated YAP1 in CRC cells interacts with transcription factor 4 (TCF4) and β-catenin to promote EMT by regulating expression of SLUG and TWIST 21. Similarly, YAP1 interacts with TEA domain transcription factor (TEAD) to promote SLUG transcription. Upregulated SLUG induces EMT in non-small cell lung cancer cells 40. In this study, we found via qPCR that YAP1 or circ1662 siRNA strongly upregulate SMAD3 expression. Rescue experiments verified this finding. We subsequently proved via qPCR, western blot, and IF experiments that YAP1 inhibits SMAD3 expression. Low SMAD3 expression was found in both TCGA and GEO data and was negatively associated with YAP1 expression. These results suggest that SMAD3 may act as a tumor suppressor in CRC. Many previous studies are consistent with our result 17, 22, 31. For example, high expression of CEACAM was found to be inversely correlated with the expression of TGF-β pathway genes, including SMAD3 in CRC. Moreover, CEACAM mutation promotes colorectal carcinogenesis by inhibiting TGF-β signaling 22. Another study proved that the tumor suppressor NIT1 blocks tumor proliferation by triggering the TGF-β1-SMAD2/3 signaling pathway in CRC 17. In this study, we found that SMAD3 siRNA promotes CRC cell migration and invasion. Moreover, SMAD3 induces E-cadherin expression. The inhibitory effects of circ1662 siRNA on CRC cell migration and invasion were reduced by SMAD3 siRNA. Based on this evidence, we conclude that circ1662 promotes EMT by regulating the YAP1-SMAD3 pathway in CRC.

circRNA formation is known to be controlled by reverse complementary sequences residing in flanking introns 8. Alu and MIRs elements are commonly reported to accelerate circRNA formation 41-43. However, we did not find any Alu elements in the reverse complementary sequences of the circ1662 upstream and downstream flanking sequences. Interestingly, its intron flanking sequences contain 10 pairs of short reverse complementary sequences that may promote circ1662 backsplicing. These findings indicate that there may be other factors that are not Alu elements that accelerate circRNA formation. RNA methylation is widely present in various types of RNA. m6A modification is one of the most common RNA methylation events. Previous studies have shown that m6A modification can regulate the biological functions of circRNAs 19, 44. YTHDF3, an m6A reader, can accelerate the efficient initiation of circRNA protein translation 44. In addition, m6A modification prevents the involvement of circRNAs in immunity. Unmodified circRNAs can activate RIG-I and innate immune signaling 19. Another interesting study verified that m6A modification promotes cytoplasmic export of circRNAs 19. These studies proved that m6A modification exists in the ring structure of circRNAs, suggesting that m6A modification could be as a regulator to affect circRNA. Early reports found that a large number of m6A modification phenomena exist in nascent RNA, and the splicing site has methylation specificity, which suggested that m6A may be involved in circRNA splicing by installation in circRNA flanking sequences 27. Surprisingly, we found abundant m6A modification in the upstream and downstream flanking sequences of circ1662. Moreover, we found that m6A modification of the circ1662 flanking repeats is dependent on METTL3. In our study, we surprisingly found that overexpressed METTL3 induced circ1662 expression in CRC cells. In contrast, silencing METTL3 decreased circ1662 expression. Taken together, these results suggest that METTL3 might regulate circ1662 formation through m6A modification.

It is well known that METTL3 is the important “writer” of m6A modification. Many studies have reported that METTL3 can act as an oncogene 28, 45, including in CRC 46. We found that METTL3 is upregulated in CRC and its high expression is correlated with poor prognosis. Moreover, METTL3 accelerates CRC cell invasion and migration. Consistent with our findings, a recent study revealed that METTL3 expedites EMT to induce metastasis in gastric cancer 47. METTL3 was reported to generate m6A, which is subsequently read by heterogeneous nuclear ribonucleoprotein A2/B1 (HNRNPA2B1) to regulate miRNA processing 48. In this study, we proved that METTL3 promoted CRC invasion and migration depending on circ1662 *in vitro*. Moreover, METTL3 accelerated CRC metastasis via the circ1662-YAP1-SMAD3 axis *in vivo*. In clinical samples, we found a strong correlation between METTL3, circ1662, YAP1, and SMAD3. These findings imply that METTL3 induces circ1662 formation to drive CRC progression.

## Conclusions

In conclusion, our data showsthat an oncogenic circRNA, circ1662, promotes CRC cell invasion and migration by enhancing EMT. Moreover, we identified that circ1662 can directly bind YAP1 and induce its nuclear transport. In addition, we reported a new regulatory mechanism of circRNA and m6A modification in which METTL3-induced circRNA splicing is dependent on m6A modification. In clinical applications, we confirmed that circ1662 could act as a prognostic biomarker and a potential target in CRC. These collective results provide new insights into diagnostic and therapeutic strategies for patients with CRC.

## Supplementary Material

Supplementary figures and tables.

## Figures and Tables

**Table 1:** Correlation analysis for clinicopathologic parameters about circ1662 expression in colorectal cancer patients

Parameters	Number of cases	Circ1662 expression		*P* value
		Low	High	
All cases	58	21	37	
**Age**				0.786
> 65	26	10	20	
≤ 65	28	11	17	
**Gender**				0.272
Male	26	7	19	
Female	32	14	18	
**Histological grade**				0.301
I-II	48	19	29	
III-IV	10	2	8	
**Vascular invasion**				0.547
Yes	3	0	3	
No	55	21	34	
**Tumor depth**				0.007
T1-T2	7	6	1	
T3-T4	51	15	36	
**Lymph node metastasis**				0.779
Yes	22	7	15	
No	36	14	22	
**Clinical stage**				0.786
1-2	34	13	21	
3-4	24	8	16	

## Abbreviations

CRC: colorectal cancer
CircRNAs: circular RNAs
EMT: epithelial to mesenchymal transition
CDH1: cadherin 1
ZO-1: tight junction protein 1
ZEB1: zinc finger E-box binding homeobox 1
SMAD3: SMAD family member 3
MIR: Mammalian-Wide Interspersed Repeats
QKI: quaking
TFs: transcription factors
UCB: urothelial carcinoma of the bladder
METTL3: methyltransferase like 3
STR: short tandem repeat
NSCLC: non-small cell lung cancer
ISH: RNA *in-situ* hybridization
FISH: fluorescence *in-situ* hybridization
IF: immunofluorescence
HE staining: hematoxylin-eosin staining
IHC: immunohistochemistry
RIP: RNA immunoprecipitation
MeRIP-qPCR: Methylated RNA immunoprecipitation qPCR
GEO: Gene Expression Omnibus
KEGG: Kyoto Encyclopedia of Genes and Genomes
ACVR2A: activin A receptor type 2A
CALM3: calmodulin 3
EGFR: epidermal growth factor receptor
HK2: hexokinase 2
SGK1: serum/glucocorticoid regulated kinase 1
SOCS3: suppressor of cytokine signaling 3
TGFBR1: transforming growth factor beta receptor 1
AJCC: American Joint Committee on Cancer
HNRNPA2B1: heterogeneous nuclear ribonucleoprotein A2/B1
TCF4: transcription factor 4
TEAD: TEA domain transcription factor
TGF-β: transforming growth factor-beta
