# Supplementary material for: N6-methyladenosine-induced circ1662 promotes metastasis of colorectal cancer by accelerating YAP1 nuclear localization
Source: Theranostics. 2021 Feb 25;11(9):4298–315. doi: 10.7150/thno.51342 (PMC7977475; doi:10.7150/thno.51342)
Supplement: Supplementary file 1 — Supplementary figures and tables. [file thnov11p4298s1.pdf]

Supplementary Tables

Table S1 The clinical data of 6 pairs CRC tissues from patients

	GDX	DX	CDZ	QXQ	XCZ	ZGT
Gender	Male	Female	Male	Male	Female	Male
Age	55	49	67	86	77	55
Tumor types	Rectal cancer	Rectal cancer	Rectal cancer	Colon cancer	Colon cancer	Rectal cancer
Pathological type	Adenocarcinoma	Adenocarcinoma	Adenocarcinoma	Adenocarcinoma	Adenocarcinoma	Adenocarcinoma
Differentiation degree	Middle	Middle	Middle	Middle	Middle	Middle
TNM stage	T2N1M0	T2N1M0	T3N2M0	T2N2M0	T3N1M0	T2N1M0
Tumor size	4*3*1	2.5*2.5*0.5	2.5*2*0.5	4.6*3.5*1.7	1.7*1.5*1.3	3*3*1
Lymph node metastasis	Yes	Yes	Yes	Yes	Yes	Yes
Distant metastasis	No	No	No	No	No	No

Table S2 The short reverse complementary sequences in circ1662 flank sequence

Score	Expect	Identities	Gaps	Strand
21.1 bits(22)	4.4	11/11(100%)	0/11(0%)	Plus/Minus

```
Query  717   ATACAGGTTTC  727
             |||||||||||
Sbjct  206   ATACAGGTTTC  196
```

Score	Expect	Identities	Gaps	Strand
21.1 bits(22)	4.4	11/11(100%)	0/11(0%)	Plus/Minus

```
Query  1634  GTATGTTGGCA  1644
             |||||||||||
Sbjct  1760  GTATGTTGGCA  1750
```

Score	Expect	Identities	Gaps	Strand
22.9 bits(24)	1.3	12/12(100%)	0/12(0%)	Plus/Minus

```
Query  1758  TAGTAAATAATT  1769
             ||||||||||||
Sbjct  2091  TAGTAAATAATT  2080
```

Score	Expect	Identities	Gaps	Strand
21.1 bits(22)	4.4	11/11(100%)	0/11(0%)	Plus/Minus

```
Query  1836  AAGTTTTTAAC  1846
             |||||||||||
Sbjct  3246  AAGTTTTTAAC  3236
```

Score	Expect	Identities	Gaps	Strand
24.7 bits(26)	0.36	16/18(89%)	0/18(0%)	Plus/Minus

```
Query  2036  ATTAAAAATATTTTAAAA  2053
             || ||| |||||||||||
Sbjct  2576  ATAAAACATATTTTAAAA  2559
```

Score	Expect	Identities	Gaps	Strand
23.8 bits(25)	1.3	14/15(93%)	0/15(0%)	Plus/Minus

```
Query  2144  CCATTTTTCTATCAT  2158
             |||||||||||| ||
Sbjct  1088  CCATTTTTCTATTAT  1074
```

Score	Expect	Identities	Gaps	Strand
21.1 bits(22)	4.4	11/11(100%)	0/11(0%)	Plus/Minus

```
Query  2447  GTAATTTGTTA  2457
             |||||||||||
Sbjct  2465  GTAATTTGTTA  2455
```

Score	Expect	Identities	Gaps	Strand
22.9 bits(24)	1.3	15/17(88%)	0/17(0%)	Plus/Minus

```
Query  2586  TGTTTATACTTAGAACA  2602
             ||| | |||||||||||
Sbjct  2640  TGTATTTACTTAGAACA  2624
```

Score	Expect	Identities	Gaps	Strand
21.1 bits(22)	4.4	11/11(100%)	0/11(0%)	Plus/Minus

```
Query  2910  GGAAATTTAAA  2920
             |||||||||||
Sbjct  1052  GGAAATTTAAA  1042
```

Score	Expect	Identities	Gaps	Strand
21.1 bits(22)	4.4	11/11(100%)	0/11(0%)	Plus/Minus

```
Query  2559  AGTAAAAAATA  2569
             |||||||||||
Sbjct  3425  AGTAAAAAATA  3415
```

Table S3 The sequence of primer, siRNA and probe

Gene	Primer-sense	Primer-antisense
circ1662	CAAGAACTGCTTCGGCAGG	GGAGAGGAATGAGCTCGAACA
circ1662	GATGAACTCGGCTTCAGCCAT	GAGACTACTCCAGTGGGGGT
YAP1	TAGCCCTGCGTAGCCAGTTA	TCATGCTTAGTCCACTGTCTGT
METTL3	GGAGTTGGGGAGAGAATG	GCGAGTGCCAGGAGATA
SMAD3	ACGACTACAGCCATTCCATCCC	TCATCTGGTGGTCACTGGTTTCT
ACVR2A	ACACAGCCCACTTCAAATCC	CAAATGACAATCCCCGCAAT
CALM3	TGGGAACGGGACCATTGACT	GACACGGAACGCCTCTCGGA
EGFR	TACTTGGAGGACCGTCGCTTG	ATGGTATTCTTTCTCTTCCGCAC
HK2	GTGCCCGCCAGAAGACATTA	TTGCTCAGACCTCGCTCCAT
MYC	AAGGCCCCCAAGGTAGTTATCC	TCGTCGTTTCCGCAACAAGTC
RELA	TACCACCAAGACCCACCCCAC	CAGCCTCATAGAAGCCATCCCG

SGK1	TTGACCCCGAGTTTACCGAA	TTGACGCTGGCTGTGACGAG
SOCS3	CCCCCCAGAAGAGCCTATTACA	TCCGACAGAGATGCTGAAGAGTG
TGFBR1	CTGTGAAGCCTTGAGAGTAATG	CCTGTTGACTGAGTTGCGATA
GAPDH	AACGGATTTGGTCGTATTGG	TTGATTTTGGAGGGATCTCG
circ1662-convergent	AGCCCTGACTCCACAGCATG	GACCAGAAGATGTCTTTGCC
circ1662-intron-upstream	GTTCTTTCACCCCTGCTC	TTAGTCATCGCTTCCCAA
circ1662-intron-downstream	ACACCTGTAATCTCAACTTCTC	ACTATGTCCTACTTTCCCCA
1663-up-03	GAGGTGGGGGTAGAACCG	GCGTTTCAGCCGACTGTAAA
1662-up-06	ACAAAAACCCGGGTTAAGGA	AACTGCTGGATATAAATTCTTGT
1662-up-07	ATTCATAAACTATTATAGTTTTGT	GTATTTACAACCTAGATTTG
1662-up-08	CACAACACAAACTGGTGACTG	AAGCCATCTACAATGAACAGGAT
1662-up-09	GAACATTTGGGCAGTTCAAA	TTAAGAGGTCAGTGCAGTTAT
1662-up-10	TAGTTGCCCTTACATGTCTA	CTATTAGGGAATATTAAGATG
1662-down-01	TAGAAACCAGCCTTCCACTT	GCAATCCACAATTCCATGTAC
1662-down-04	AGAAGGTACTGTTGGTTTT	AAGAAACTCATAGTTATTTAGGC
1662-down-06	TAGGGCTGTATTTTAAAAGTATC	TGGAGTATCATTTTCTGAGC
1662-down-07	ATGGTTCTTTGACAAACTAAG	GGTTAGTAAATAATTCTCTTTGG
1662-down-09	TATCTGACATAAAAAAGGATG	AAGAACTTTTTTTTTAGTTTC
1662-down-11	CATACTAATAGACTATTTTTAG	CATGGTCTGGCTGTGTAGG

siRNA	Target sequence
si-circ1662-01	GCCACAGGCCAGTACTGAT
si-circ1662-02	CCACAGGCCAGTACTGATG
si-YAP1	CAGTGGCACCTATCACTCT
si-METTL3	GCTGCACTTCAGACGAATT
si-SMAD3	GAGGCTGTCTACCAGTTGA

Gene	Probe sequence
Circ1662(ISH)	5‘-DIG-ATCAGTACTGGCCTGTGGCCTCACCTG-DIG-3’
Circ1662(FISH)	5’ Cy3-AGTACTGGCCTGTGGCCTCACCT
Circ1662(Pull down)	5’-ATCAGTAC+TGGCCTGTGGCC+TCACCTGC-3’
NC (Pull down)	5’-GCTACGCAGCCGTCTCTCGTATGTACCG-3’

Supplementary figures

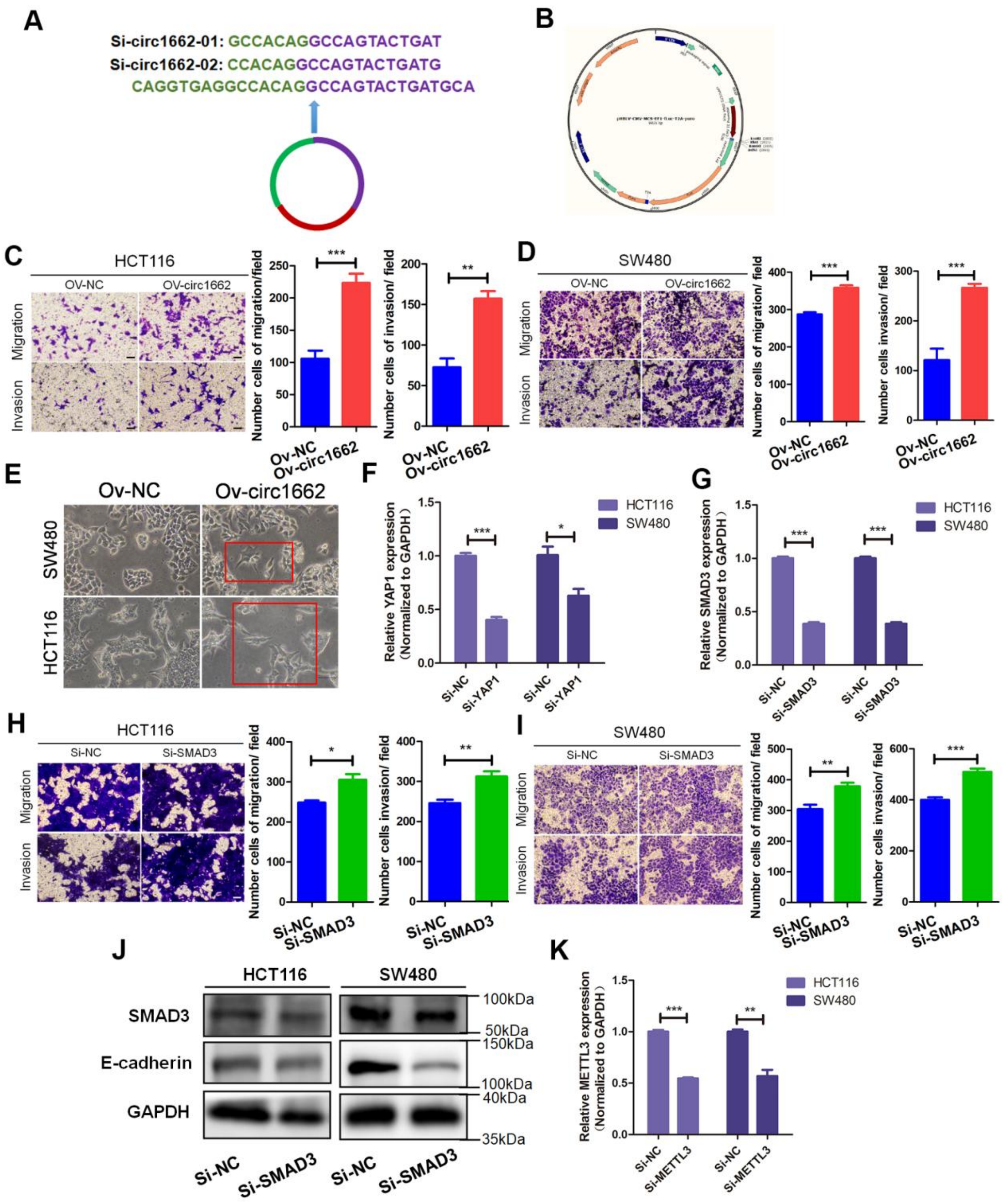

Supplementary Figure 1. (A) The design of si-circ1662 which was across the junction site. (B) Overexpressed vector of circ1662. (C, D) Transwell assay of HCT116 and SW480 transfected circ1662 vector for evaluating migration and invasion. The number of migration and invasion was counted in HCT116 and SW480. (E) The cell morphology of HCT116 and SW480 transfected circ1662 overexpression plasmids after 72h. The effect of YAP1 (F) and SMAD3 (G) siRNA in HCT116 and SW480 using qPCR analysis. (H, I) Transwell assay of HCT116 and SW480 transfected SMAD3 siRNA. The number of migration and invasion was counted in HCT116 and SW480. (J) Western blot analysis of SMAD3 and E-cadherin in HCT116 and SW480 transfected SMAD3 siRNA. (K) The effect of METTL3 siRNA in HCT116 and SW480 using qPCR analysis. GAPDH is the negative control. Student's t test was use to determined the *P* value (*P < 0.05, 0.001 < ** P < 0.01, *** P < 0.001), Mean ± SEM.

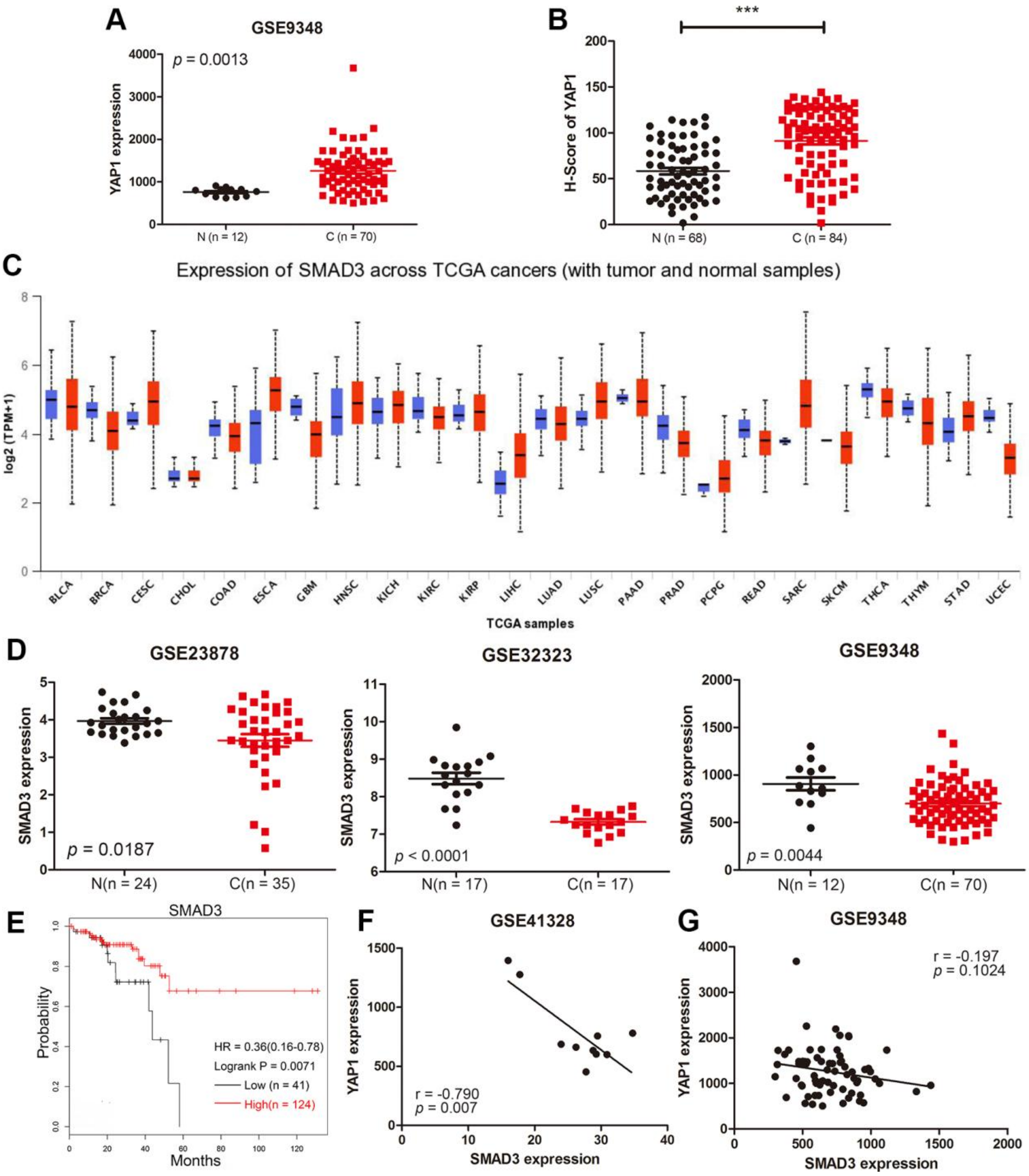

Supplementary Figure 2. (A) GEO data (GSE9348) analyzing SMAD3 expression in CRC tissues (n = 12) compared normal tissues (n = 70). (B) H-score of YAP1 in 54 pairs CRC tissuea compared to normal tissues from tissue chip. (C) UALCAN showing the pan-cancer of SMAD3 expression which data from TCGA. (D) GEO data (GSE23878) analyzing SMAD3 expression in CRC tissues (n = 35) compared normal tissues (n = 24). GEO data (GSE32323) analyzing SMAD3 expression in CRC tissues (n = 17) compared normal tissues (n = 17). GEO data (GSE9348) analyzing SMAD3 expression in CRC tissues (n = 12) compared normal tissues (n = 70). (E) Survival analysis of high SMAD3 expression compared to low SMAD3 expression in rectal cancer via KM-plotter online tool. Then it was assessed by log-rank test. (F, G) GEO data (GSE41328, GSE9348) analyzing the correlation between YAP1 and SMAD3 in CRC tissues. Student's t test was use to determined the *P* value (**P* < 0.05, 0.001 < ** *P* < 0.01, *** *P* < 0.001), Mean ± SEM.

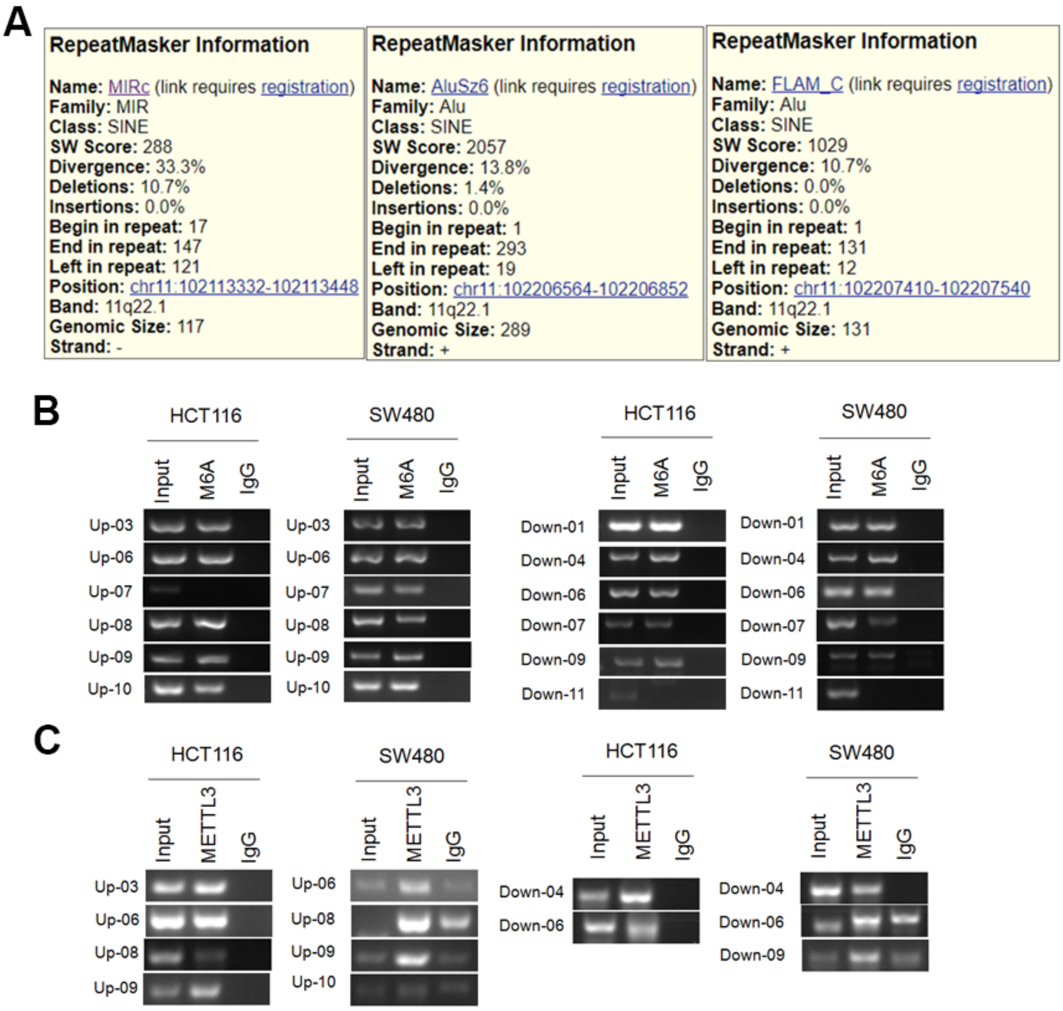

Supplementary Figure 3. The squence alignment and qPCR production identified by agarose gel electrophoresis. (A) The up-and down flank sequences of circ1662 were aligned in the UCSC Genome Browser. MIRc element, MIR family; AluSz6 and FLAM C elements, Alu family. (b) Circ1662-up-03, 06, 07, 08, 09, 10 and circ1662-down-01, 04, 06, 07, 09, 11 in HCT116 and SW480 were detected by MeRIP-qPCR. (C) Circ1662-up-03, 06, 08, 09 and circ1662-down-04, 06 were pulled down by METTL3 antibody using RIP-qPCR in HCT116. Circ1662-up-06, 08, 09, 10 and circ1662-down-04, 06, 09 were pulled down by METTL3 antibody using RIP-qPCR in SW480. Input group was the positive control and IgG group was the negative control.

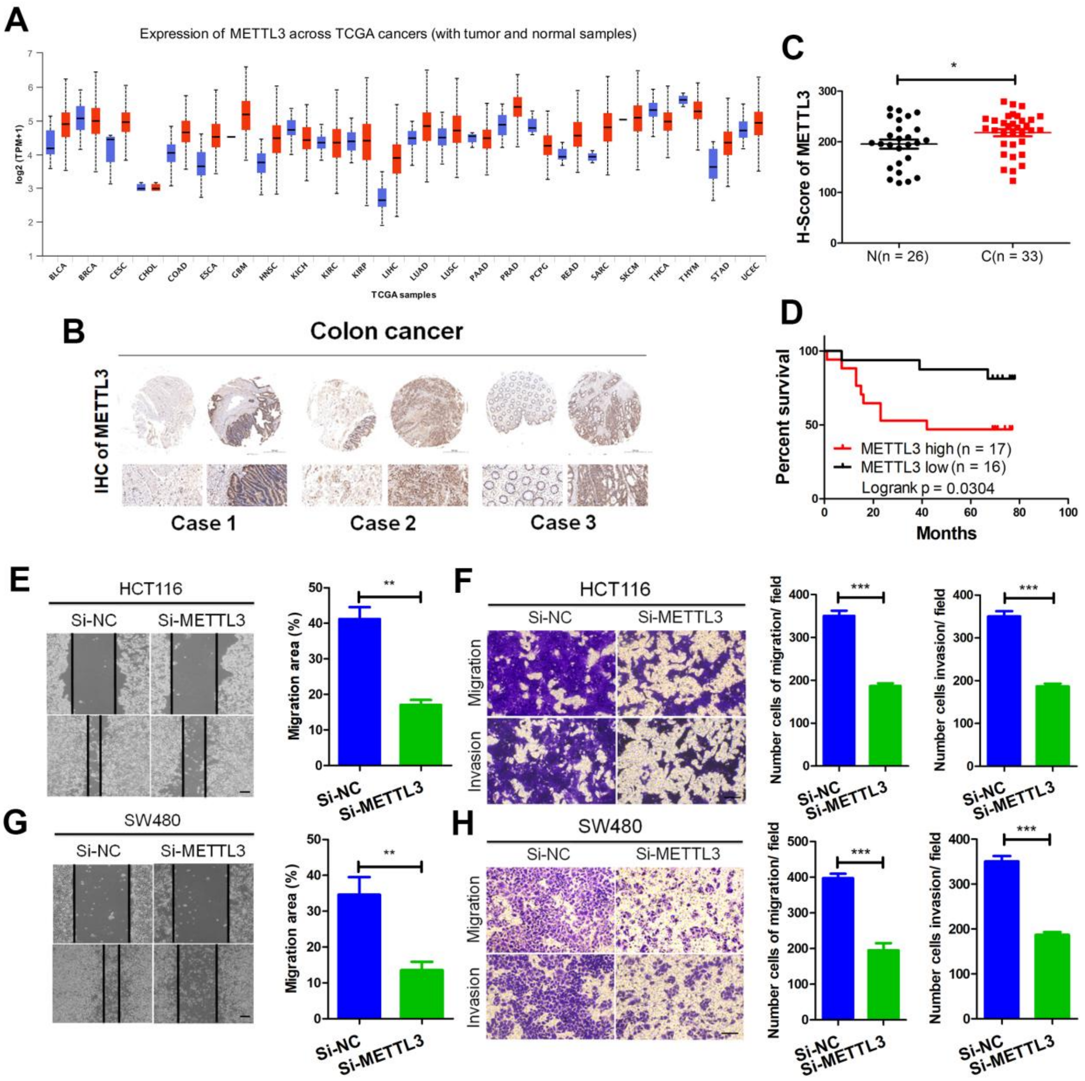

Supplementary Figure 4. The role of METTL3 in CRC (A) UALCAN showing the pan-cancer of METTL3 expression which data from TCGA. (B) IHC image of METTL3 expression in CRC tissue chip. (C) H-score of METTL3 in CRC tissues (n = 26) compared to normal tissues (n = 33) from tissue chip. (D) Survival analysis of high METTL3 expression compared to low METTL3 expression in CRC tissues from CRC tissue chip. Then it was assessed by log-rank (Mantel-Cox) test. (E, F) The migrated and invasive ability of HCT116 transfected METTL3 siRNA using wound healing assays and transwell assays. The migration area was counted by image J. (G, H) The migrated and invasive ability of SW480 transfected METTL3 siRNA using wound healing assays and transwell assays. The migration area was counted by image J. Statistical significance was calculated by Student's t test, **P* < 0.05, 0.001 < ** *P* < 0.01, *** *P* < 0.001, Mean ± SEM.
